# Loss of the fructose transporter SLC2A5 inhibits cancer cell migration

**DOI:** 10.3389/fcell.2022.896297

**Published:** 2022-09-30

**Authors:** Jody Groenendyk, Konstantin Stoletov, Tautvydas Paskevicius, Wenjuan Li, Ning Dai, Myriam Pujol, Erin Busaan, Hoi Hei Ng, Aristeidis E. Boukouris, Bruno Saleme, Alois Haromy, Kaisa Cui, Miao Hu, Yanan Yan, Rui Zhang, Evangelos Michelakis, Xing-Zhen Chen, John D. Lewis, Jingfeng Tang, Luis B. Agellon, Marek Michalak

**Affiliations:** ^1^ Department of Biochemistry, University of Alberta, Edmonton, AB, Canada; ^2^ Department of Oncology, University of Alberta, Edmonton, AB, Canada; ^3^ Department of Medicine, University of Alberta, Edmonton, AB, Canada; ^4^ Wuxi Cancer Institute, Affiliated Hospital of Jiangnan University, Wuxi, Jiangsu, China; ^5^ National “111” Center for Cellular Regulation and Molecular Pharmaceutics, Hubei University of Technology, Wuhan, China; ^6^ Department of Physiology, University of Alberta, Edmonton, AB, Canada; ^7^ School of Human Nutrition, McGill University, Montreal, QC, Canada

**Keywords:** metastasis, cancer, mitochondria, invadopodia, cell migration

## Abstract

Metastasis is the primary cause of cancer patient death and the elevation of SLC2A5 gene expression is often observed in metastatic cancer cells. Here we evaluated the importance of SLC2A5 in cancer cell motility by silencing its gene. We discovered that CRISPR/Cas9-mediated inactivation of the SLC2A5 gene inhibited cancer cell proliferation and migration *in vitro* as well as metastases *in vivo* in several animal models. Moreover, SLC2A5-attenuated cancer cells exhibited dramatic alterations in mitochondrial architecture and localization, uncovering the importance of SLC2A5 in directing mitochondrial function for cancer cell motility and migration. The direct association of increased abundance of SLC2A5 in cancer cells with metastatic risk in several types of cancers identifies SLC2A5 as an important therapeutic target to reduce or prevent cancer metastasis.

## Introduction

Metastasis, which is defined as the development of secondary tumors, remains the major cause of death for patients with cancer (Steeg, 2006). The process of metastasis depends on enhanced cancer cell motility, proliferation, and subsequent colonization of a new microenvironment. Melanomas and pancreatic cancers are particularly metastatic if not caught early during the course of the disease (Chaffer & Weinberg, 2011; Budczies et al., 2015).

Fructose is transported across membranes by SLC2A5/GLUT5, a member of the facilitative glucose transporter family. SLC2A5 was first identified in the intestine (Douard & Ferraris, 2008), and exhibits high specificity for fructose, and does not transport glucose nor galactose (Uldry & Thorens, 2004). Notably, increased abundance of SLC2A5 mRNA and protein in cancer cells is associated with cancer progression, increased frequency of metastasis, and an unfavorable prognosis for many cancers (Zamora-Leon et al., 1996; Chen et al., 2016; Bu et al., 2018; Hamann et al., 2018; Weng et al., 2018; Jin et al., 2019; Lin et al., 2021). SLC2A5 expression is higher in metastatic liver lesions than in normal liver; and elevated in primary lung tumors; as well as in brain, colon, testis, and uterine cancers, including breast carcinoma cell lines (Uldry & Thorens, 2004). Moreover, recent studies have shown that fructose serves as an efficient energy source for lung cancer cells (Chen et al., 2020) and facilitates tumor cell proliferation (Liang et al., 2021).

Many strategies that target metastasis have been studied and explored (Steeg, 2016), however, the role SLC2A5 plays in promoting metastasis and metastatic progression remains unknown. Here we show that silencing of the SLC2A5 fructose transporter by gene editing in cancer cells inhibited cell motility and proliferation *in vitro* as well as inhibited cancer cell invasion and metastasis in chicken embryo, mouse, and zebrafish models of human cancer progression. Moreover, attenuation of SLC2A5 function caused a reduction in the number of mitochondria, alteration of their morphology as well as their localization in cancer cells, underscoring the importance of mitochondrial function in cell motility.

### Experimental procedures

Ethics. All methods were carried out in accordance with relevant guidelines and regulations and approved by Biosafety Officers in the Department of Environment, Health and Safety, at the University of Alberta. All animal experiments were carried out according to the University of Alberta Animal Policy and Welfare Committee and the Canadian Council on Animal Care Guidelines. The approval for use of mice in research was granted by the Animal Care and Use Committee for Health Sciences, a University of Alberta ethics review committee (Permit AUP297).

CRISPR/Cas9 Gene Editing and Cell Culture. MIA-PaCa-2 (ATCC# CRM-CRL-1420), MDA-MB-231 (ATCC# CRM-HTB-26), HeLa (ATCC# CRM-CCL-2) were obtained from ATCC (Manassas, VA) and HT1080 (ATCC# CCL-121) through Thermo Fisher Scientific. MIA-PaCa-2 cancer cells were isolated from a male patient (Yunis et al., 1977). HT1080 fibrosarcoma cells, originally isolated from a male patient (Rasheed et al., 1974), expressing the red fluorescent protein tdTomato (cells referred to hereafter as HT1080tdT) (Leong et al., 2014) were used in this study. For CRISPR/Cas9 gene editing, the sequence of the guided RNA was identified using the WTSI Genome Editing website (www.sanger.ac.uk). The two following ssDNA guide oligonucleotides were targeted to Exon 3 of human SLC2A5: 5′-GATCCGATAAACCCTCCAAA-3′; 5′-GAAGTCTTCCATGAATTCAC-3′. The ssDNA guide oligonucleotides were annealed with their complement and cloned into the PX459 plasmid containing the gRNA scaffold and the Cas9 nickase. Cells for targeted gene editing were grown under standard culturing conditions (5% CO_2_; 37°C) and standard cell culture medium (DMEM; Gibco Cat# 11995073) plus 10% fetal bovine serum. Positive clones were selected with puromycin for 24 h and seeded onto a 96 well plate to generate single cell colonies. Clones were cultured and genomic DNA harvested using the DNeasy Blood and Tissue kit (Qiagen) with genomic DNA utilized for PCR, with the PCR product gel purified (Qiagen) and sequenced using SLC2A5 specific primers. Positive clones were identified by aligning with the SLC2A5 gene sequence. Clones carrying an edited SLC2A5 allele (identified with the suffix -ε2A5) were determined to have 62 base pairs removed, leading to an early stop codon 14 amino acids after the second oligonucleotide sequence. Off-target effects were monitored by sequencing ALDH1b1 and HADHB genes, which were identified as potential off-target genes.

Full-length cDNA encoding human SLC2A5 was subcloned into the pCMV6 expression vector using restriction enzymes AsiSI (also called Sgf1) and MluI using following DNA primers: SLC2A5-Sgf1 forward primer: 5′-CGC​GCG​ATC​GCA​TGG​AGC​AAC​AGG​ATC​AGA​G-3′ and SLC2A5-Mlu1 reverse primer: 5′-CGCACGCGTCTG​TTC​CGA​AGT​GAC​AGG​TG-3′. SLC2A5 E401A mutant was generated using Q5^®^ High-Fidelity 2X Master Mix (New England Biolabs). Primers used were as follows: SLC2A5-E401A forward primer, 5′-GCT​CAT​CAC​TGC​TAT​CTT​CCT​GCA​GTC​CTC-3′, and SLC2A5-E401A reverse primer, 5′-AGC​GCG​GGT​ATG​GGA​CTG-3′. Cells were transfected with the assembled expression vector encoding either human wild-type SLC2A5 or the non-functional SLC2A5-E401A mutant (Nomura et al., 2015). Scratch test, proliferation assay and mitochondria imaging were carried out after the times indicated in the Figure legend.

Immunoblot Analysis and Flow Cytometry. Protein assay, SDS-PAGE, and immunoblotting analysis were carried out as described (Groenendyk et al., 2014). Anti-β-tubulin antibodies were from Thermo Fisher (Cat# MA5-16308) and used at a dilution of 1:2000; anti-GAPDH antibodies (Cat# Ab8245) and anti-mitofusin-1 antibodies (Cat# ab129154) were purchased from Abcam and used at the dilution of 1:1000. Anti-SLC2A5 antibodies were purchased from Boster (Cat# PA 2064) and used at 1:500 dilution. Anti-Flag tag antibodies were from Thermo Fisher (Cat# 740001) and used at 1:100 dilution.

Secondary antibodies, IRDye^®^ 680RD donkey anti-mouse IgG secondary (Cat# 925–68072) and IRDye^®^ 800CW donkey anti-rabbit IgG secondary antibodies (Cat# 926–32212) were from LI-COR and were used at a dilution of 1:10000. Densitometry was performed using ImageJ software (NIH) and plotted in Excel. For Flow Cytometry analysis MIA-PaCa-ε2A5 or MIA-PaCa-ε2A5 transfected with expression vector encoding wild-type SLC2A5 or SLC2A5 E401A mutant were grown to 80% confluency and harvested with TrypLE (Thermo Fisher). Cells were pelleted and incubated in the Fixation Buffer (Invitrogen) for 10 min followed by pelleting and permeabilization with Permeabilization Buffer (Invitrogen) for 10 min. To block non-specific interactions, cells were pelleted and incubated for 30 min in the Permeabilization Buffer containing 1% BSA. Cells were incubated at 4°C for 16 h with mouse anti-Flag antibodies (1:100 dilution; Pierce, Cat# MA1-91878) in Permeabilization Buffer containing 1% BSA. Cells were washed 3 times with Permeabilization Buffer containing 1% BSA followed by addition of anti-mouse Alexa Fluor 488 secondary antibody (1:100 dilution; Thermo Fisher), washed 3 times with Permeabilization Buffer containing 1% BSA and resuspended in PBS for Flow Cytometry using an LSRFortessa™ X-20 Flow cytometer (BD Biosciences Pharmingen). Data analysis was performed using FlowJo™ vX for PC (TreeStar).

qPCR Analysis. RNA was isolated from MIA-PaCa-2 and MIA-PaCa-ε2A5 cells, HT1080tdT cells and SLC2A5-deficient HT1080tdT-ε2A5 cells using the RNeasy Kit (Qiagen) according to the manufacturer’s protocol. Quantitative PCR (qPCR) analysis was used for determination of the mRNA abundance using a RotorGene Q rapid thermal cycler system (GE Life Sciences) according to the manufacturer’s instructions. Total RNA (200 ng) was used to synthesize cDNA (BioRad) according to manufacturer’s protocol then the resulting cDNA was diluted 5-fold, and 2 μL of the diluted sample was used in qPCR reactions with primers targeting the mRNA of interest (see below). qPCR reactions were conducted in duplicate on three separate occasions. The Ct values for selected targets were normalized to the Ct value of glyceraldehyde 3-phosphate dehydrogenase (GAPDH). The following primers were used for qPCR analyses:

GAPDH 5′-AAT​GTG​TCC​GTC​GTG​GAT​CTG​A-3′; 5′-AGT​GTA​GCC​CAA​GAT​GCC​CTT​C-3′

NRF1 5′- GCC​ACA​GCC​ACA​CAT​AGT​ATA​G-3′; 5′- CGT​ACC​AAC​CTG​GAT​AAG​TGA​G-3′

SLC2A1 5′-GTG​CTC​CTG​GTT​CTG​TTC​TT-3′; 5′-CTC​GGG​TGT​CTT​GTC​ACT​TT-3′

SLC2A2 5′-GGG​ACT​TGT​GCT​GCT​GAA​TA-3′; 5′-CCT​GGC​CCA​ATT​TCA​AAG​AAG-3′

SLC2A4 5′-CTG​GAC​GAG​CAA​CTT​CAT​CA-33′; 5′-CAG​GAG​GAC​CGC​AAA​TAG​AA-3′

SLC2A5 (targeted to Exon 12) 5′- CCT​CAC​CAC​CAT​CTA​CAT​CTT​C-3′, 5′-GGG​TAC​ACT​TCA​GAC​ACC​TTA​TT-3′

ALU 5′-GGT​GAA​ACC​CCG​TCT​CTA​CT-3′; 5′-GGT​TCA​AGC​GAT​TCT​CCT​GC-3′

MFN1 5′-GGG​CCC​TAG​AAA​TGC​TCA​AA-3′; 5′-GCA​GTG​GGA​GTA​GAA​GCT​AAA​G-3′

NCAD 5′-GAC​AGT​TCC​TGA​GGG​ATC​AAA​G-3′; 5′-CGA​TTC​TGT​ACC​TCA​ACA​TCC​C-3′

VIM 5′-GCT​GTG​GAT​GTG​AGG​TGA​GC-3′; 5′-GCT​AAA​ATC​AAG​GCA​AAC​CCT​AAG​TC-3′.

Scratch Test, Proliferation Assay, and Colony Formation Assay. Scratch tests were performed with MIA-PaCa-2 and SLC2A5-deficient MIA-PaCa-ε2A5 cells, HT1080tdT and HT1080tdT-ε2A5 cells, MDA-MB-231 and MDA-MB-231-ε2A5, HeLa and HeLa-ε2A5 cells. Cells were plated at equal cell numbers in a 12-well dish and grown to confluency. The cell layer was scratched with a P200 pipet tip straight down the middle of the well. The wells were washed with 2 ml of phosphate-buffered saline (PBS) and then fresh cell culture medium was added. Each scratch was photographed at times indicated in the Figure legend. The width of the scratch was measured at three locations along the length of the scratch using ImageJ then the results were compiled and analyzed in GraphPad Prism.

Cell viability was assessed using the 3-(4,5-dimethylthiazol-2-yl)-5-(3-carboxymethoxyphenyl)-2-(4-sulfophenyl)-2H-tetrazolium (MTS) assay. Cells (1 × 10^3^ or 2 × 10^3^) were seeded in triplicate into 96-well plates and grown in standard culture medium. The next day, the growth medium was replaced with fresh standard culture medium supplemented with MTS (1:10 dilution of a 10% MTS) (Promega; Cat# G3582), incubated for 2 h at 37°C, then samples of the culture medium was taken from each well for determination of absorbance at 490 nm was measured using a 96-well plate reader.

MIA-PaCa-2 and MIA-PaCa-ε2A5 (clone B3 or F11) cells were plated at a density of 1.0 × 10^4^ cells/well into 96-well plates and maintained in regular growth medium. HT1080tdT and HT1080tdT-ε2A5 cells were plated at a density of 5.0 × 10^3^ cells/well into 96 well plates and maintained in regular growth medium. Fructose was added to the media at 1 and 10 mM concentrations and MIA-PaCa-2 and MIA-PaCa-ε2A5 cells (clones B3 or F11) were incubated for 48 h and HT1080tdT and HT1080tdT-ε2A5 for 24 h. Cell proliferation in the absence or presence of added fructose was determined using the MTS assay as described above.

For soft agar colony assays, cells (3 × 10^3^) were plated in triplicate in 0.35% low melting point agarose layered on top of 0.7% agarose in 6-well plates and covered with standard culture medium (Chen et al., 2016). Colonies were counted and photographed after 2 weeks. For the colony formation assay, cells (1 × 10^3^) were seeded in triplicate into 6-well plates and grown in standard culture medium. Colonies were fixed with 70% ethanol and stained with 1% trypan blue (Sigma-Aldrich) (Figure 3B), rinsed with water and dried. Plates were photographed, colonies counted and analyzed by the OpenCFU software. For agarose colony assay (Supplementary Figure S3) MIA-PaCa-2 and MIA-PaCa-ε2A5 cell lines (1 × 10^4^ cells/well) were mixed with 0.6% agarose in normal growth media and plated on top of the 1% agarose layer (Weng et al., 2018). Cells were grown for 3 weeks with media changed every 3 days. Plates were photographed and colonies counted and analyzed by OpenCFU software. Experiments were performed on at least three independent occasions.

Transmigration Assay. Cell migration was assayed in 24-well Transwell plates (8.0-μm pore size; BD Pharmingen). Cells (1 × 10^4^ cells/insert) were cultured for 24 h on the upper side of the filter with 100 µl of serum-free medium. The lower chambers were filled with 700 µl of DMEM supplemented with 10% FBS. After 24 h, non-migrating cancer cells on the upper surface of the membrane were removed using a Q-tip^®^ and migrated cancer cells on the undersides of the Transwell membranes were fixed with cold methanol then stained for 60 min with a solution containing 1% Coomassie brilliant blue dye, 50% methanol, 10% glacial acetic acid. The cell chamber was washed 3 times with 45% methanol and 10% glacial acetic acid, dried, and photographed. The cell number was determined by counting cells using ImageJ. Cells that dropped from the membrane to the well below were stained with Alamar Blue according to the manufacturer’s protocol (Thermo Fisher Scientific).

FITC gelatin degradation assay. FITC gelatin degradation assay was performed as previously described (https://bio-protocol.org/e997). Briefly, FITC gelatin coated 18 mm cover slides were prepared using glutaraldehyde fixation and dropped into 12 well tissue culture plates. HT1080tdT or HT1080tdT-ε2A5 cells (2 × 10^4^) were seeded on the top of the FITC gelatin coated 18 mm cover slides and incubated for 10 h. Cells were fixed using formaldehyde and stained with Alexa Fluor 568 Phalloidin (to detect actin) and DAPI (to visualize nuclei). Images were acquired using Nikon A1 confocal microscope and analyzed using ImageJ. Data are displayed as degraded FITC gelatin area fraction (arbitrary units). Experiments were performed in triplicates.

Electron Microscopy and Confocal Imaging. Transmission electron microscopy experiments were performed by plating MIA-PaCa-2 and MIA-PaCa-ε2A5 cells on special coverslips with a Cu-300 mesh (Maxtaform) for use in a Hitachi HT-7650 electron microscope with a bottom mount AMT camera (4864 × 3264 pixel fixed bottom). Cells were only allowed to grow to 30% confluency to prevent crowding. Coverslips were fixed, negative stained, and then imaged using a Hitachi H-7650 transmission electron microscope at 60 kV. Twenty images (3,000x magnification) were taken of two samples for each cell line. Mitochondria were hand traced and the mitochondria length and diameter were determined using ImageJ. Confocal imaging was performed on living cell cultures for each cell line as previously described (Prins et al., 2011).

Staining and Quantitation of Mitochondria. MitoTracker^®^ Green FM mitochondria staining was carried out as described by manufacturer (Invitrogen). Briefly, a working solution containing 10 nM MitoTracker^®^ Green FM and 0.1 μg/ml in a standard complete medium prewarmed to 37°C was applied to live cells cultured in a 4-well Nunc Lab Tek chamber slide. Chamber slides were sealed, and cells were allowed to recover at 37°C for 30 min. Mitochondria were visualized using Nikon A1 microscope equipped with 10x (for localization) or 63× (for number and length) objectives. Mitochondria length was quantified manually using Nikon Elements length measurement module.

*Ex Vivo* Chick Embryo Cancer Xenograft Model and Mouse Spontaneous Metastasis Model. Fertilized White Leghorn chicken eggs were purchased from the University of Alberta Poultry Research Centre and incubated in a humidified chamber at 38°C. At Day 4, embryos were removed from their shells using a cutting wheel and maintained in a covered dish at 38°C and 60% humidity. On Day 10 of development, the chicken embryos were injected intravenously with 2.5 × 10^4^ of HT1080tdT or HT1080tdT-ε2A5 cells, and metastatic colonies were allowed to grow for 6 days. For time lapse imaging of metastatic colonies, sterilized coverslips were applied on top of the embryos that contained metastatic colonies by 24 h post tumor cell application.

The tumor xenograft study in mice was carried out in 8-week-old female BALB/c-nude mice housed in a pathogen-free environment. HT1080tdT cells or HT1080tdT-ε2A5 cells (2 × 10^6^) were injected subcutaneously in the bilateral rear flank. After 5 days of injection, the tumor volumes were measured every day. Mice were euthanized after 12 days of cell injection. The xenograft tumors were removed for weighing and photographing, and the lungs were taken to observe metastasis using 2-photon microscopy (Zeiss) as previously described (Stoletov et al., 2018). For the microscopic imaging of mouse lungs, freshly excised lungs were washed twice with PBS and placed under the cover glass and into the humidified chamber. Nikon A1 microscope equipped with 10× objective was used for imaging.

To quantify metastatic HT1080-derived cancer cells, the murine lung tissue was removed, followed by extraction of genomic DNA (Stoletov et al., 2018). Metastasis was quantified using qPCR with primers directed at human Alu elements and normalized to the mouse GAPDH gene copy number (Stoletov et al., 2018). Mouse lung tissue was collected, homogenized in sterile sample tubes and genomic DNA was extracted using SYBR^®^ Green Extract-N-Amp Tissue PCR Kit (Sigma-Aldrich). Human Alu element sequences were detected and quantified by qPCR (PCR profile: 95°C for 3 min followed by 40 cycles of 95°C for 30 s, 60°C for 30 s, 72°C for 30 s). Data are displayed as 2ΔΔCt.

Injection of Cancer Cells into Zebrafish Embryos. Two days post fertilization transgenic zebrafish embryos Tg (fli1:EGFP) were anesthetized using 1.2 mM tricaine and transferred to a Petri dish coated with 1% agarose. Fibrosarcoma cells were trypsinized and collected as a single cell suspension in a 15 ml collection tube. After 5 min of centrifugation at 6,000 *g*, the supernatant was removed, and the pellet was resuspended in PBS. After a second centrifugation, the supernatant was removed. The pellet was resuspended in 20 μL 2% polyvinylpyrrolidone (PVP) in PBS and held at room temperature before implantation. Approximately 300 HT1080tdT cells or HT1080tdt-ε2A5 cells in a volume of 5 nL were injected into the pre-cardiac sinus of zebrafish embryos using the microinjector (PV820, World Precision Instruments). After the injection, the embryos were removed from the agarose plate, placed into the 10 cm Petri dish containing fresh water and incubated at 33°C for 3 and 5 days. Three days post injection embryos were randomly selected for fluorescent imaging. Fluorescent images were taken using a LEICA M165 FC stereo fluorescence microscope. Confocal imaging was performed at 5 dpi using a LEICA SP8 confocal microscope. Prior to confocal imaging, zebrafish embryos were immobilized using 1.2 mM tricaine and then embedded into a thin layer of low-melting point agarose to maintain the fish in a lateral position. Fluorescent and confocal images were analyzed using ImageJ software.

Statistical analysis. Statistical analysis was performed using GraphPad Prism with a Student’s t-test used to compare the mean of two independent groups or one-way ANOVA used to compare the mean of three or more independent groups with the difference determined to be significant if the *p* < 0.05.

## Results

Attenuation of SLC2A5 function inhibits cancer cell migration. Increased expression of SLC2A5 has been associated with disease progression, increased metastasis, and an unfavorable prognosis of several types of cancers including cervical cancer, renal carcinoma, lung carcinoma, hepatocellular carcinoma, endometrial cancer cells, and pancreatic cancer (Zamora-Leon et al., 1996; Chen et al., 2016; Bu et al., 2018; Hamann et al., 2018; Weng et al., 2018; Jin et al., 2019; Lin et al., 2021) (Supplementary Figure S1A). Increased abundance of SLC2A5 is also associated with reduced survival in pancreatic adenocarcinoma and hepatocellular carcinoma (Supplementary Figure S1B).

**FIGURE 1:**
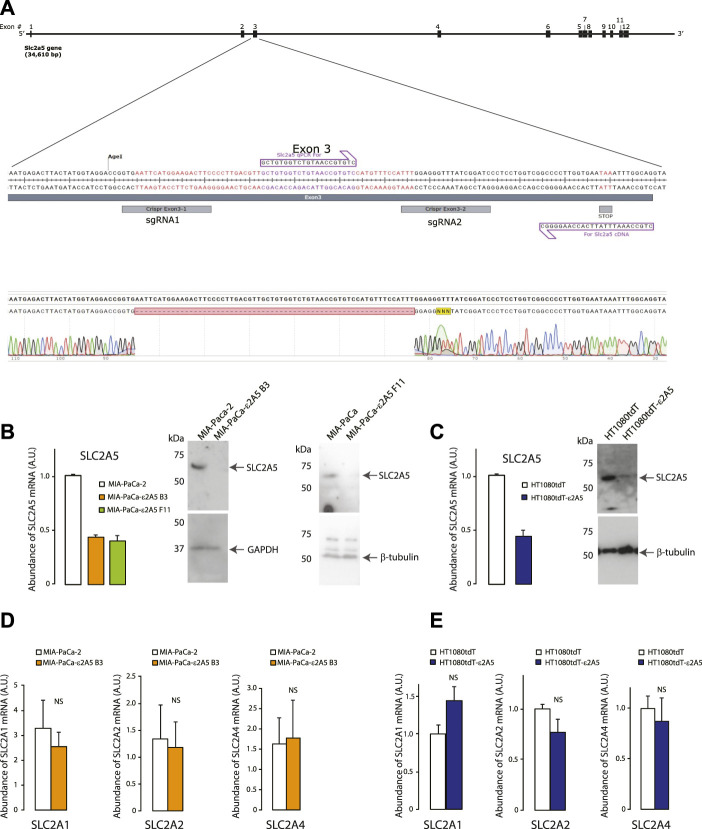
Editing of the SLC2A5 gene. **(A)**. Inactivation of the SLC2A5 gene in human MIA-PaCa-2 and HT1080tdT cells using CRISPR/Cas9-mediated *in situ* gene editing. Guide RNAs were targeted to Exon 3. Genomic DNA of edited cells was sequenced, and positive clones were identified as having a 62 base pair disruption, leading to an early stop codon 14 amino acids downstream of the disruption point. **(B)**. qPCR analysis of SLC2A5 mRNA abundance in MIA-PaCa-2 and MIA-PaCa-ε2A5 cells (clones B3 and F11) (n = 3). DNA primers were designed to amplify Exon 12 of the SLC2A5 gene. Immunoblot analysis was carried out with antibodies directed at the C-terminus of SLC2A5. Anti-β-tubulin or anti-GAPDH antibodies were used to assess amounts of loaded protein samples. **(C)**. qPCR analysis of SLC2A5 mRNA abundance in HT1080tdT and HT1080tdT-ε2A5 cells. DNA primers and control antibodies were as described above. **(D)**. qPCR analyses of SLC2A1, SLC2A2, and SLC2A4 mRNA abundance in MIA-PaCa-2 and MIA-PaCa-ε2A5 cells (n = 3). **(E)**. qPCR analyses of SLC2A1, SLC2A2, and SLC2A4 mRNA abundance in HT1080tdT and HT1080tdT-ε2A5 cells (n = 3). All data in the Figure is representative of more than 3 biological replicates. NS, not significant.

To test directly if deletion of the SLC2A5 gene impacts cancer progression and migration, we used CRISPR/Cas9 gene editing to inactivate the SLC2A5 gene in a highly metastatic pancreatic ductal adenocarcinoma MIA-PaCa-2 cell line, and fibrosarcoma HT1080tdT cells (Figure 1A). Editing of the SLC2A5 gene in MIA-PaCa-2 cells (hereafter referred to as MIA-PaCa-ε2A5 cells; Figure 1B) or HT1080tdT cells (hereafter referred to as HT1080tdT-ε2A5 cells; Figure 1C) had no effect on the abundance of mRNA encoding other members of the SLC2 family of solute transporters, namely SLC2A1, SLC2A2, SLC2A4 (Figures 1D,E). Editing of the SLC2A5 gene also did not alter the expression of N-cadherin and vimentin, markers of the mesenchymal phenotype (Supplementary Figure S2).

**FIGURE 2:**
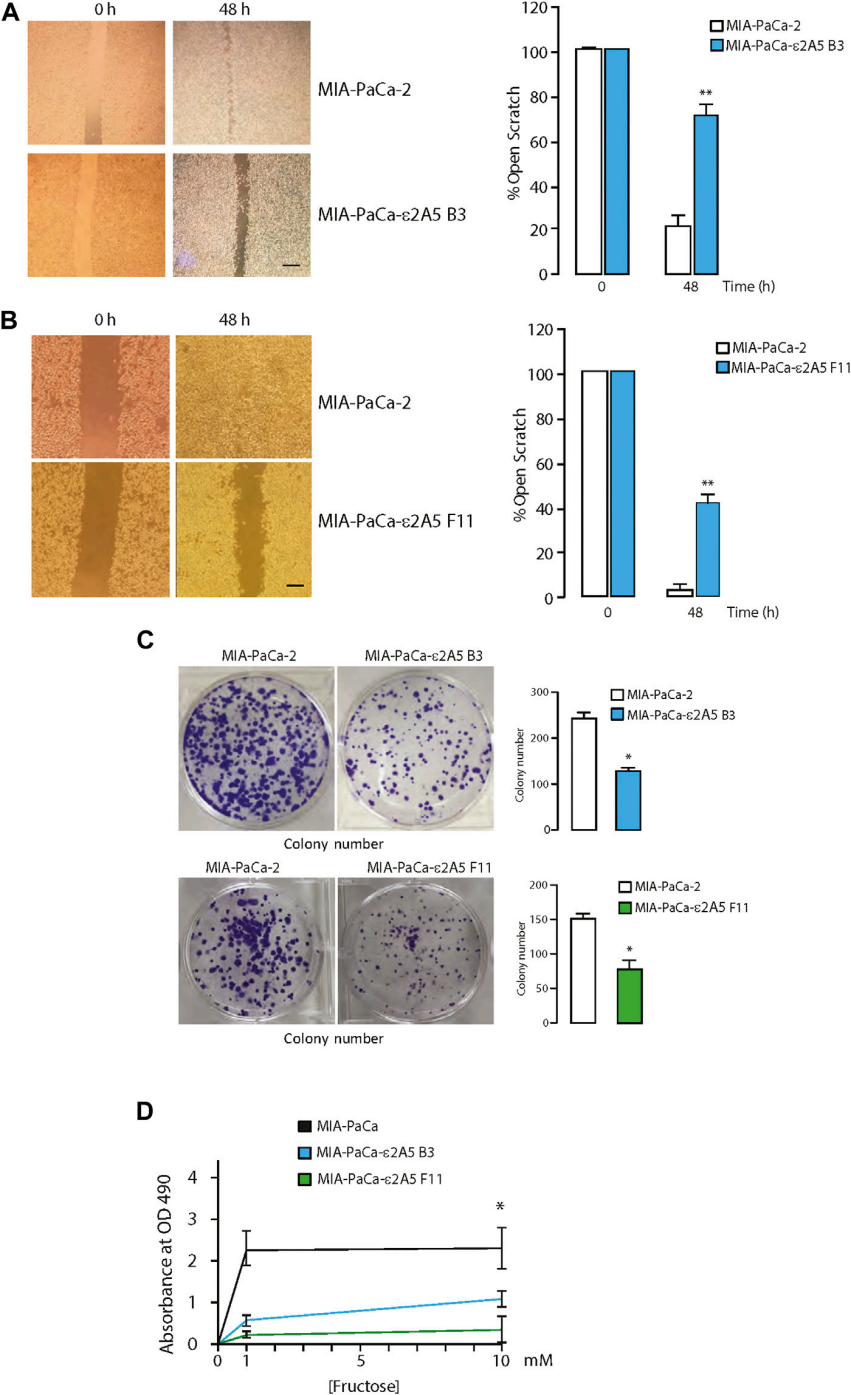
Attenuation of the SLC2A5 gene expression in pancreatic cancer cells. **(A)**. Images of scratch test (left panel) and quantitative analysis of closure (right panel) for MIA-PaCa-2 and MIA-PaCa-ε2A5 cells (clone B3; **p = 0.002, n = 3). Scale bar 10 µm. **(B)**. Scratch test analysis for MIA-PaCa-2 and MIA-PaCa-ε2A5 cell lines clone F11 (**p = 0.001, n = 3). Scale bar 10 µm. **(C)**. Colony formation images and colony number for MIA-PaCa-ε2A5 cells, clone B3 (left panel; 1000 cells/well plated at time 0 h; *p = 0.0001; n = 3) and clone F11 (right panel; 500 cells/well plated at time 0 h; *p = 0.001; n = 3). **(D)**. Fructose-dependent proliferation of the MIA-PaCa-2 and MIA-PaCa-ε2A5 cells. MIA-PaCa-2 and MIA-PaCa-ε2A5 (clones B3 and F11) cells were plated at a density of 1.0 × 10^4^ cells/well of a 96 well plates. Fructose was added to the culture medium as indicated in the figure. The absorbance at 490 nm (OD_490_) of the culture medium after MTS assay at time 0 and 48 h in culture are shown. Experiments were performed a minimum of three times in triplicates. *p = 0.0002; n = 3.

Two independent clones of MIA-PaCa-2 cells with edited SLC2A5 genes (referred to as clones MIA-PaCa-ε2A5 B3 and MIA-PaCa-ε2A5 F11) were established (Figure 1B). First, MIA-PaCa-2 and MIA-PaCa-ε2A5 cells were grown to confluency and subjected to a scratch test assay. After 48 h, only a small portion of the scratch remained for MIA-PaCa-2 cells (Figures 2A,B). Strikingly, the majority of the scratch remained for MIA-PaCa-ε2A5 cells d since they were unable to migrate efficiently during the same time period (MIA-PaCa-ε2A5 clone B3, Figure 2A and MIA-PaCa-ε2A5 clone F11, Figure 2B). Furthermore, both clones of MIA-PaCa-ε2A5 cells exhibited reduced average colony count (Figure 2C) and reduced proliferation (Figure 2D) compared to MIA-PaCa-2 cells. Addition of fructose to the culture medium of MIA-PaCa-2 cells increased proliferation (Figure 2D) whereas neither clone of MIA-PaCa-ε2A5 responded (Figure 2D). As well, MIA-PaCa-ε2A5 cells showed inhibited colony formation in the soft agar transformation assay (Supplementary Figure S3) (Borowicz et al., 2014). We also used CRISPR/Cas9 gene editing to silence the SLC2A5 gene in HeLa cervical cancer cells (Supplementary Figure S4A) and in breast cancer MDA-MB-231 cells (Supplementary Figure S4B). Similar to MIA-PaCa-ε2A5 cells (Figure 2B), attenuated SLC2A5 gene expression in HeLa-ε2A5 or MDA-MB-231-ε2A5 cells rendered these cells unable to close the scratch by 48 h (Supplementary Figure S4).

**FIGURE 3:**
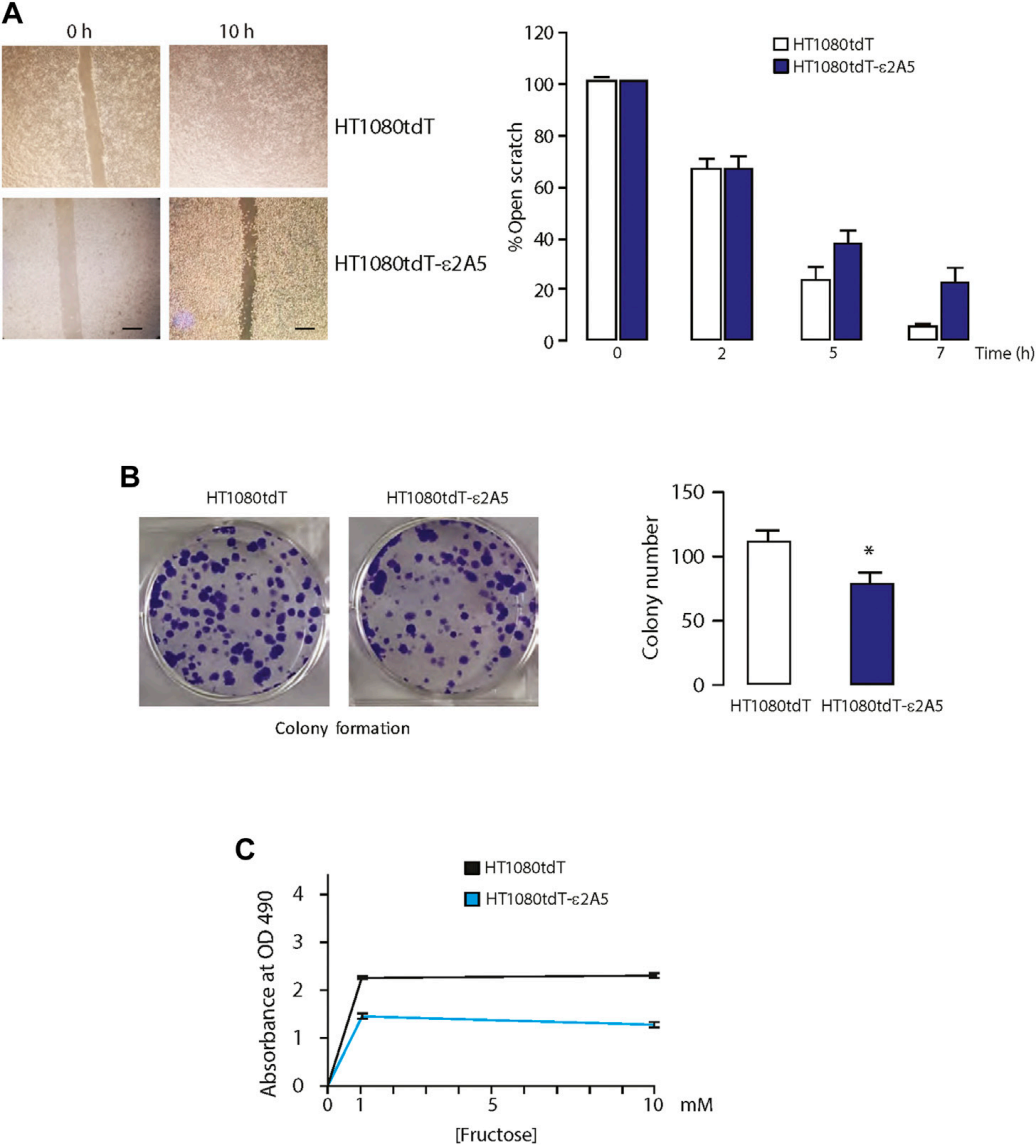
Attenuation of the SLC2A5 gene expression in fibrosarcoma cells. **(A)**. Images of scratch test (left) and quantitative analysis of closure (right) as a function of time for HT1080tdT and HT1080tdT-ε2A5 fibrosarcoma cells (n = 3). Scale bar 10 µm. **(B)**. Images of colonies formed by HT1080tdT and HT1080tdT-ε2A5 cells and analysis of colony number (500 cells/well plated at time 0 h). *p = 0.007; n = 3. Colonies were fixed with 70% ethanol and visualized by staining with 1% trypan blue. **(C)**. Proliferation assay of HT1080tdT and HT1080tdT-ε2A5 cells. HT1080tdT and HT1080tdT-ε2A5 cells were plated at a density of 5.0 × 10^3^ cells/well of a 96 well plates. Fructose was added to the media as indicated in the Figure. Cells were cultured for 48 h followed by MTS assay (n = 3). All data in the Figure is representative of more than 3 experiments with 3 biological replicates.

**FIGURE 4:**
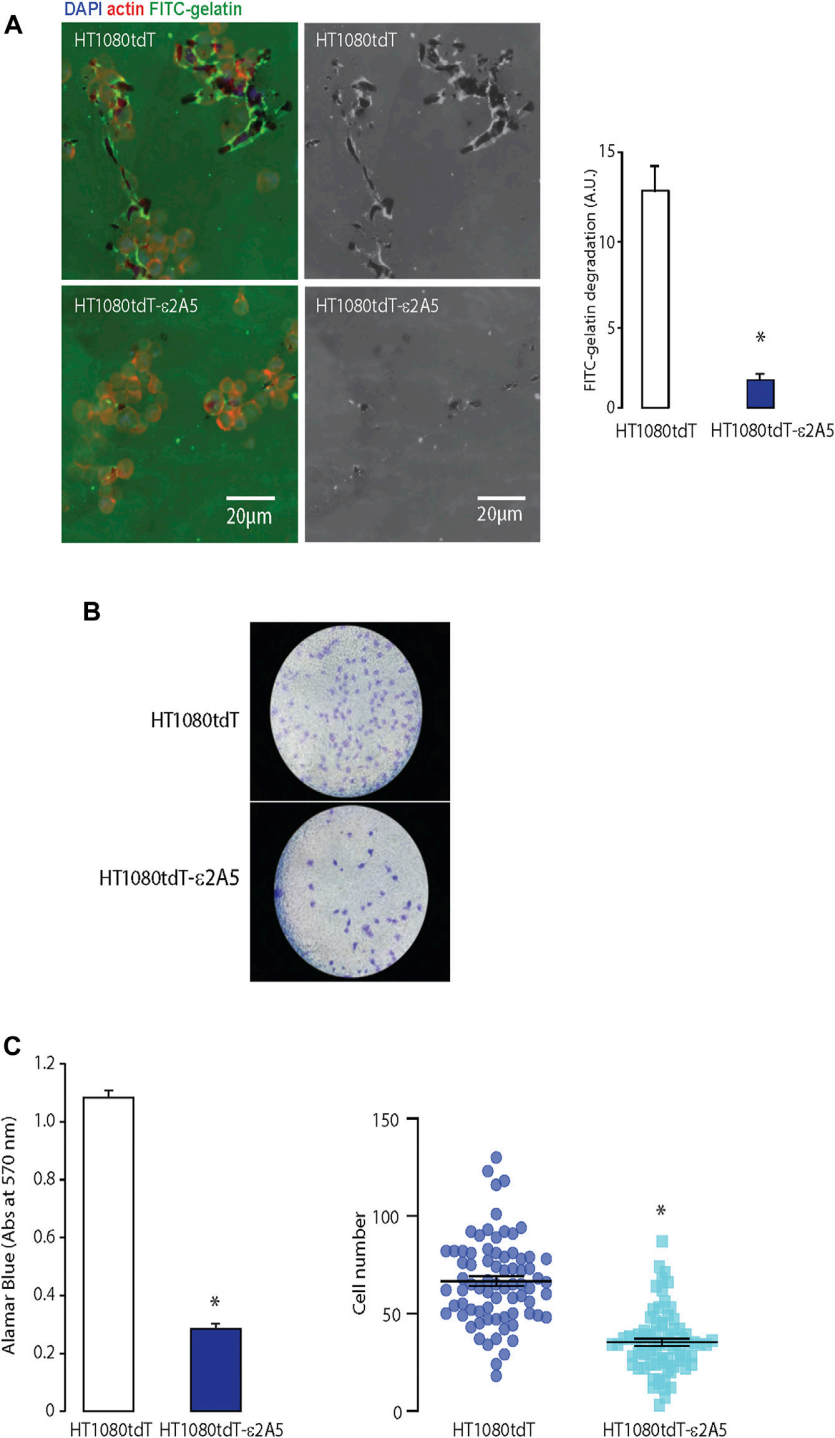
Migration of the HT1080tdT-ε2A5 fibrosarcoma cells. **(A)**. An FITC-gelatin degradation assay for invasiveness of HT1080tdT and HT1080tdT-ε2A5 cells. Samples were co-stained with DAPI and actin. *p < 0.0001, n = 3. **(B)**. Transwell migration of HT1080tdT and HT1080tdT-ε2A5. Cells were fixed in 100% methanol and stained with Coomassie blue (n = 3). **(C)**. Transmigration of HT1080tdT and HT1080tdT-ε2A5 across an 8 µm pore membrane. *p < 0.0001, n = 3. The images are representative of more than 3 biological replicates.

The HT1080tdT fibrosarcoma cells display higher metastatic potential than MIA-PaCa-2. Similar to MIA-PaCa-ε2A5 cells, HT1080tdT cells with attenuated SLC2A5 gene expression (HT1080tdT-ε2A5) were also delayed in closing the scratch (Figure 3A). In addition, these cells showed a 20% reduction in colony formation on plastic compared to the HT1080tdT cells (Figure 3B). Proliferation of HT1080tdT-ε2A5 cells was reduced, and as expected, in response to fructose supplementation (Figure 3C). Interestingly, although we observed reduced proliferation of HT1080tdT-ε2A5 compared to HT1080tdT in response to fructose supplementation, the difference was not statistically significant (Figure 3C). Next, we carried out a series of experiments to analyze SLC2A5 function in cell invasion and migration using a FITC-gelatin degradation assay. Compared to HT1080tdT, HT1080tdT-ε2A5 cells exhibited reduced invasiveness (Figure 4A). In transmigration experiments, HT1080tdT-ε2A5 cells were unable to migrate efficiently across an 8 µm pore membrane, and had a reduced ability to drop to the lower chamber (Figures 4B,C). These results demonstrate that limiting SLC2A5 gene expression in a variety of cancer cells has an inhibitory effect not only on proliferation but also on cell migration.

**FIGURE 5:**
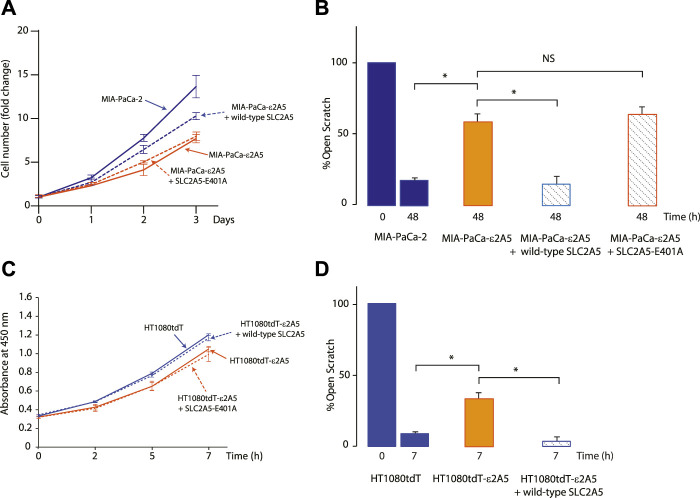
Restoration of SLC2A5 function via SLC2A5 transgene expression. Proliferation assay **(A)** and scratch test assay **(B)** of MIA-PaCa-2, MIA-PaCa-ε2A5 (clone F11) and MIA-PaCa-ε2A5 cells expressing wild-type SLC2A5 or the non-functional SLC2A5-E401A mutant (Nomura et al., 2015) (*p = 0.002; n = 3). In **(A)**, fold change in cell number is shown at day 0 to day 3. In **(B)**, the percentage of open scratch is shown at time 0 and 48 h. Proliferation assay **(C)** and scratch test assay **(D)** of HT1080tdT, HT1080tdT-ε2A5 cells transfected with expression vectors encoding wild-type SLC2A5 or the non-functional SLC2A5-E401A mutant (*p = 0.001, n = 3). In **(C)**, the absorbance at 490 nm (OD_490_) of the culture medium after MTS assay from time 0 and 7 h in culture is shown. In **(D)**, the percentage of open scratch is shown at time 0 and 7 h. NS, not significant. All data in the Figure is representative of more than 3 experiments with 3 replicates.

To determine if the alterations in cellular proliferation and migration of SLC2A5 gene-edited cells were caused by the inhibition of the SLC2A5 gene, expression vectors encoding wild-type or mutant SLC2A5 were created and introduced into MIA-PaCa-ε2A5 and HT1080tdT-ε2A5 cells. Trans-expression of wild-type SLC2A5 restored cellular proliferation of MIA-PaCa-ε2A5 and HT1080tdT-ε2A5 and motility increased to a level similar to that seen for the unedited cell lines (Figures 5A–D, respectively). The glutamic acid residue at position 400 of the rat SLC2A5 protein (corresponds to E401 in the human SLC2A5 protein) forms a critical inter-bundle salt bridge with the rat E151 (E152 in human SLC2A5) to enable fructose binding and transport, and replacement of this glutamic acid residue with alanine reduces d-fructose binding to the transporter by 90% (Nomura et al., 2015). To test whether fructose binding/transport activity is involved in the enhanced motility of cancer cells, we introduced the E401A mutant of the human SLC2A5 into SLC2A5 gene-edited MIA-PaCa-2 or HT1080tdT cells. Expression of the E401A SLC2A5 mutant in MIA-PaCa-ε2A5 cells or HT1080tdT-ε2A5 had no effect on cell proliferation (solid vs. dashed orange lines, Figures 5A,C, respectively) nor motility of MIA-PaCa-ε2A5 cells in the scratch test (Figure 5B). FACS analysis of MIA-PaCa-ε2A5 cells transfected with transgenes encoding Flag-tagged wild-type or E401A mutant of SLC2A5 confirmed the production and cell membrane localization of the re-introduced SLC2A5 proteins (Supplementary Figure S5). Collectively, these trans complementation experiments demonstrated that enhanced proliferation and motility of MIA-PaCa-2 and HT1080tdT cells require the full activity of wild-type SLC2A5.

**FIGURE 6:**
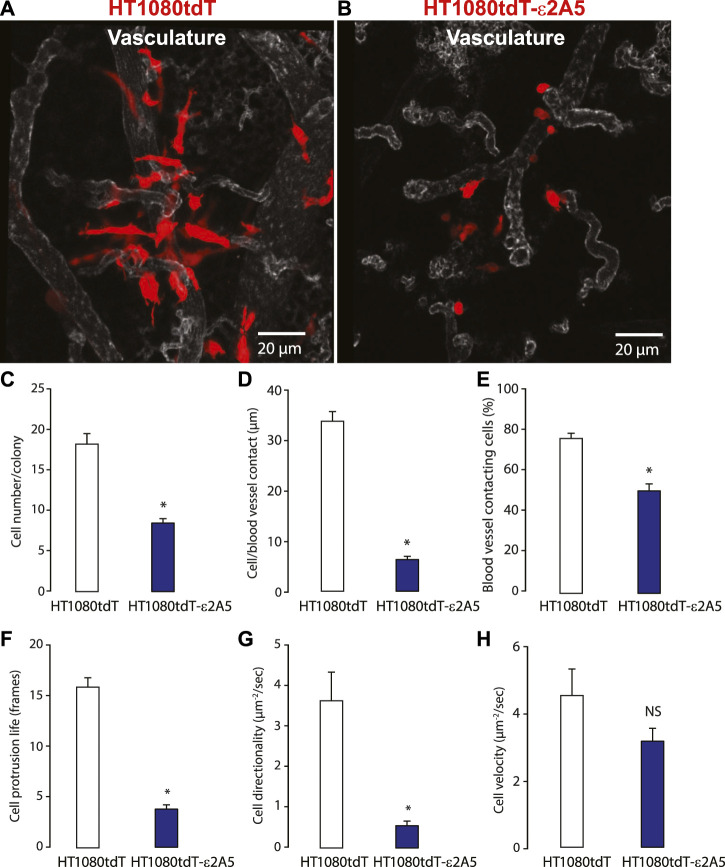
Analysis of metastatic behaviour of the HT1080tdT and HT1080tdT-ε2A5 using the chicken embryo chorioallantoic membrane (CAM) technique. **(A)** and **(B)**. Images of HT1080tdT **(A)** and HT1080tdT-ε2A5 cells **(B)** in chicken embryo CAM vasculature. The corresponding time-lapse video is shown in the Supplementary Video S1. **(C–H)** Quantitative analyses of cell number per colony **(C)**; cell to blood vessel contacts **(D,E)**; cell protrusion **(F)**; cell directionality and cell velocity **(H)**. *p < 0.0001; n = 3; NS, not significant. The images shown are representative of more than 3 biological replicates.

**FIGURE 7:**
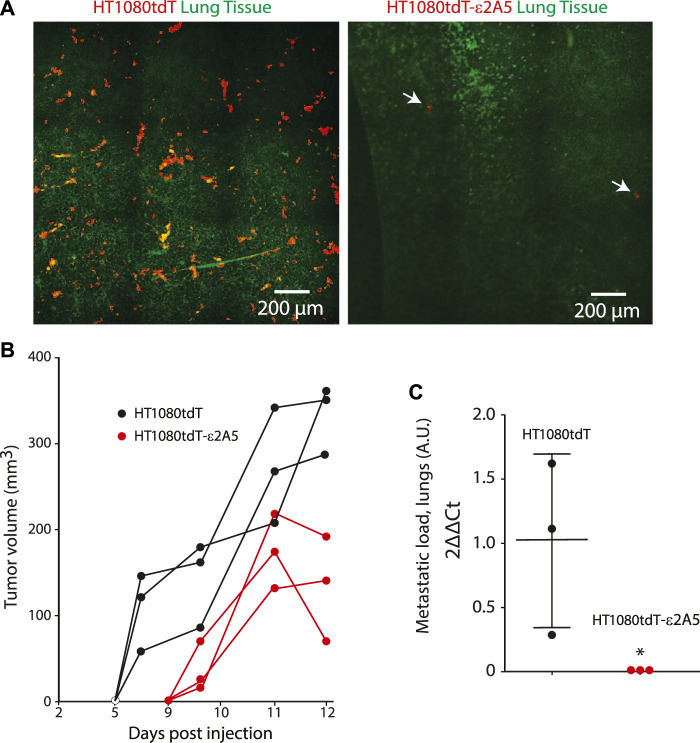
Xenograft murine model of spontaneous metastasis. **(A)**. Fluorescence stereomicroscopy images of lungs from mice bearing tumors derived from fibrosarcoma HT1080tdT cells or HT1080tdT-ε2A5 cells injected into the flank of nude mice. HT1080-derived cells are visible as red cells due to stable expression of the tdTomato transgene (Leong et al., 2014). Arrows indicate the location of two HT1080tdT-ε2A5 cells identified in lung of mice injected with HT1080tdT-ε2A5 cells. **(B)**. Reduced tumor volume in HT1080tdT-ε2A5 injected mice (n = 3). **(C)**. Quantitation of the metastatic lesions, as identified by presence of human Alu elements, formed by the HT1080tdT and HT1080tdT-ε2A5cells. *p < 0.0001, n = 3.

**FIGURE 8:**
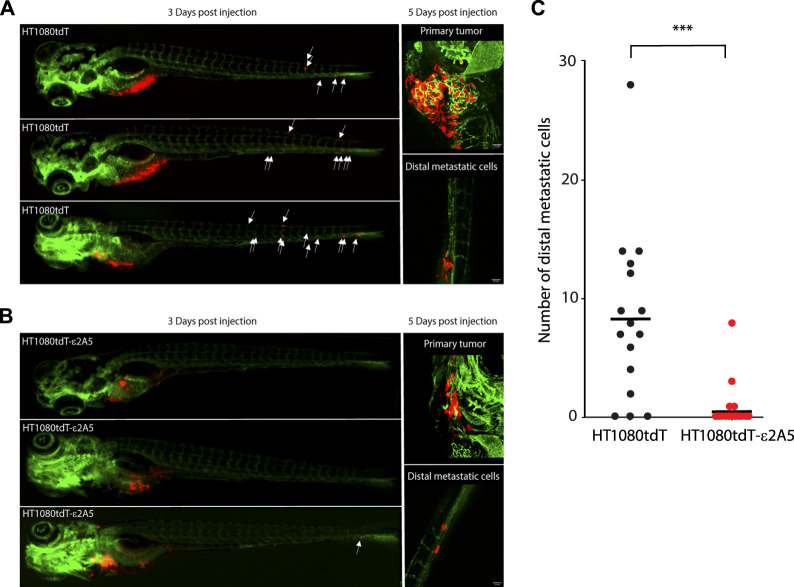
HT1080tdT cells extravasation in zebrafish embryos. **(A)** and **(B)**. Representative fluorescent (left) and confocal (right) images of transgenic zebrafish embryos Tg (fli1:EGFP) injected with HT1080tdT cells **(A)** or HT1080tdT-ε2A5 cells **(B)**. Fluorescent images were taken 3 days post injection, whereas confocal images were taken 5 days post injection. White arrows indicate the position of migrated HT1080tdT cells. **(C)**. Quantification of migrated HT1080tdT cancer cells at 3 days post injection; HT1080tdT, n = 16; HT1080tdT-ε2A5, n = 24, ***p < 0.0001. The images in the figure are representative of more than 3 biological replicates.

Inhibition of SLC2A5 limits HT1080tdT fibrosarcoma cancer cell invasion and metastasis *in vivo*. We used three animal models to evaluate the importance of SLC2A5 in cancer metastasis *in vivo*: the chicken embryo chorioallantoic membrane (CAM) (Willetts et al., 2016), xenograft murine model and the zebrafish. For these experiments, we used HT1080-derived cells due to the highly metastatic nature of the parental cell line (Stoletov et al., 2007). First, in the chicken CAM model, intravenous injection of red fluorescent protein labelled HT1080tdT fibrosarcoma cells robustly formed colonies within 3–5 days, and these cells formed extended contacts with the CAM vasculature (Figure 6A and Supplementary Video S1). Strikingly, attenuation of the SLC2A5 gene expression led to decreased contacts with the CAM vasculature (Figure 6B) and ∼50% reduction in metastatic colony size (Figure 6C). Moreover, HT1080tdT-ε2A5 cells displayed rounded morphology with a significant decrease both in cancer cell-blood vessel wall contact length and number of blood vessel contacting cells (Figures 6D,E). Furthermore, intravital time-lapse analysis of HT1080tdT-ε2A5 cells showed markedly less cell directionality, and often changed their movement direction (Figures 6F,G). However, attenuation of the SLC2A5 gene expression had no apparent influence on cancer cell migration velocity (Figure 6H). Second, we employed a xenograft murine model of spontaneous metastasis to the lungs to test for HT1080 metastasis. Human HT1080tdT cells or HT1080tdT-ε2A5 cells were injected into the flank of nude mice, followed by monitoring of tumor formation and lung metastasis using quantitative PCR assay of human Alu elements and confocal microscopy. As expected, HT1080tdT cells robustly metastasized and formed multicellular metastatic lesions with visible protrusions extending into the mouse lung tissue as compared to HT1080tdT-ε2A5 (Figure 7A). In contrast, HT1080tdT-ε2A5 cells grew slower and the metastatic lesions formed by the HT1080tdT-ε2A5 cells were reduced in volume and comprised of fewer cells (Figures 7B,C). Third, we used the zebrafish to assess the ability of HT1080tdT cells to survive in the blood circulation and establish secondary metastatic colonies. HT1080tdT or HT1080tdT-ε2A5 cells were injected into the pericardium of transgenic zebrafish that express GFP throughout their vasculature and then analyzed 3–5 days after injection. Zebrafish that were injected with HT1080tdT cells developed large tumors as well as metastatic lesions in the tail segments (Figures 8A,B) whereas the zebrafish injected with HT1080tdT-ε2A5 cells developed fewer and smaller tumors (Figure 8C). Collectively, these results illustrate that decreased SLC2A5 function resulted in substantially impaired efficiency of HT1080 cells to form metastases *in vivo*.

**FIGURE 9:**
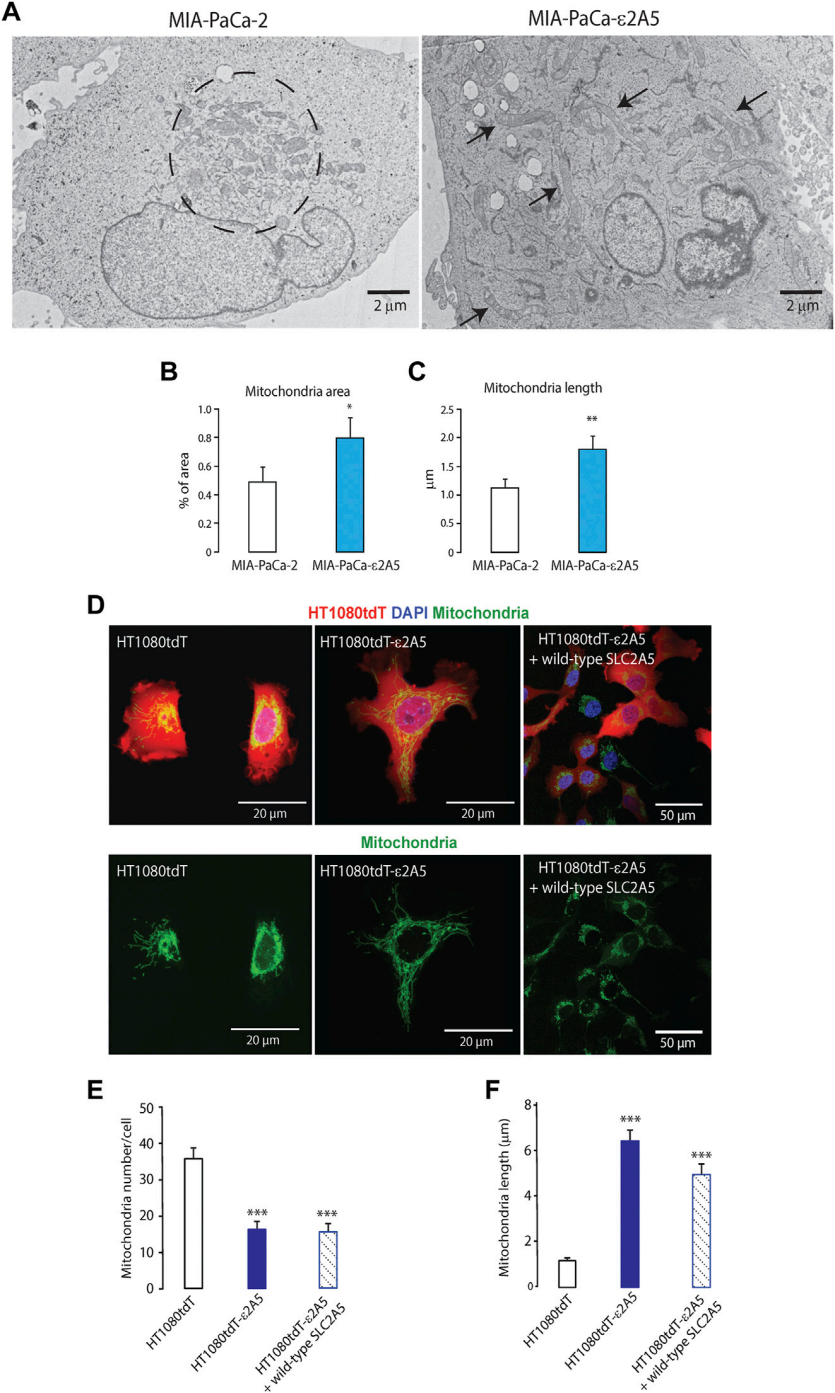
Mitochondria localization and dynamics in SLC2A5-deficient cancer cells. **(A)**. Electron microscopy analysis of MIA-PaCa-2 cells shows that mitochondria were localized in the center of these cells in the perinuclear space (encircled). In MIA-PaCa-ε2A5 cells, mitochondria were spread throughout the cell towards the cell periphery, and appeared elongated (arrows) (n = 3). **(B)**. and **(C)**. Mitochondria area **(B)** and length **(C)** analyses in MIA-PaCa-2 and MIA-PaCa-ε2A5 cells. *p < 0.0006; **p < 0.0001 (n = 3). **(D)**. Confocal images of mitochondria stained with MitoTracker^®^ Green in red fluorescent protein-labelled HT1080tdT cells,HT1080tdT-ε2A5 cells or HT1080tdT-ε2A5 cells expressing wild-type SLC2A5. Time-lapse video is shown in the Supplementary Video S2 (arrowhead). **(E)**. Mitochondria number and **(F)**. mitochondria length in fibrosarcoma HT1080tdT cells, HT1080tdT-ε2A5 or HT1080tdT-ε2A5 cells expressing wild-type SLC2A5. ***p < 0.0002 (n = 3).

Reduced SLC2A5 function alters distribution and morphology of mitochondria in cancer cells. Mitochondrial dynamics are linked to cancer cell migration (Desai et al., 2013; Zhao et al., 2013; Landry et al., 2014; Schuler et al., 2017; Sun et al., 2018; Denisenko et al., 2019; Furnish & Caino, 2020). Thus, we analyzed the impact of SLC2A5 inhibition on mitochondrial localization and morphology in MIA-PaCa-2 and HT1080tdT cells. In MIA-PaCa-2 cells, electron microscopy analysis showed clusters of mitochondria localized adjacent to the nucleus in parental cells (Figure 9A, encircled in the left image). In MIA-PaCa-ε2A5 cells, however, the mitochondria were dispersed throughout the cell body (Figure 9A, right), and increased in both total surface area and length (elongated) (Figures 9B,C). We carried out qPCR and immunoblot analyses of the abundance of MFN-1 (mitofusin-1) mRNA and protein, a mediator of mitochondria fusion (Legros et al., 2002). Although the MFN-1 mRNA abundance in MIA-PaCa-ε2A5 cells only tended to be higher compared to MIA-PaCa-2 cells (Supplementary Figure S6A), the MFN-1 protein abundance was clearly increased as a consequence of SLC2A5 attenuation (Supplementary Figure S6B).

**FIGURE 10:**
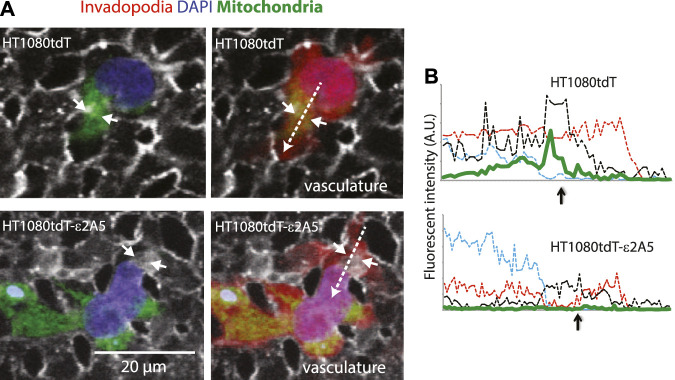
SLC2A5 function is necessary for polarized localization of mitochondria in HT1080tdT human fibrosarcoma cells *in vitro* and *in vivo*. **(A)** mitochondria (green) localization in HT1080tdT or HT1080tdT-ε2A5 (vasculature, grey; blue, DAPI) cells that are extravasating out of the chicken CAM vasculature (*in vivo*). Right panels show all three channels: red fluorescent protein (red), mitochondria (green), nuclei (blue) and vasculature (grey). Time-lapse video is shown in the Supplementary Video S3. **(B)**. Fluorescent channel intensity along the line scans (dashed arrows in **(A)** (right panel) indicate the direction of the scan). Green line depicts mitochondria localization. The short arrows in **(A)** and **(B)** indicate vascular membrane breaches.

Analysis of HT1080tdT-ε2A5 cells revealed a similar pattern of changes in mitochondrial distribution and morphology as seen in MIA-PaCa-ε2A5 cells (Figure 9D). Mitochondria in HT1080tdT-ε2A5 cells were also dispersed throughout the cell (Figure 9D), decreased in number (Figure 9E) and became elongated (Figure 9F). Importantly however, trans-expression of wild-type SLC2A5 in HT1080tdT-ε2A5 cells restored the perinuclear distribution as seen in HT1080tdT cells (Figure 9D). In the time-lapse video of HT1080tdT cells grown in cell culture, we observed mitochondrial trafficking from the central cell body to the leading edge of cells migrating toward each other (Supplementary Video S2, arrowhead). In contrast, HT1080tdT-ε2A5 cells showed loss of directional movement of mitochondria, and the mitochondria remained dispersed throughout the cell body (Supplementary Video S2). Cancer cell extravasation drives tumor cell protrusions across the endothelium from the vessel lumen into tissue and it is a key step in cancer metastasis (Strilic & Offermanns, 2017). Therefore, we injected HT1080 cells into the chicken CAM vasculature to observe extravasation. Extravasating HT1080tdT cells exhibited prominent concentration of mitochondria in the leading invadopodium (Figures 10A,B, upper panels). Time-lapse video of extravasating HT1080tdT cells revealed directional movement of mitochondria towards the leading edge of migrating cells (Supplementary Video S3). In contrast, HT1080tdT-ε2A5 cells caused both dispersed localization and loss of directional movement of mitochondria, and formation of multiple invadopodia pointing to random directions (Figures 10A,B, lower panels). Migration of HT1080tdT-ε2A5 cells also lacked defined directionality (Supplementary Video S3). Importantly, the loss of SLC2A5 resulted in attenuation of extravasation *in vivo* (Figures 10A,B and Supplementary Video S3). Taken together, our data showed that SLC2A5 function is necessary for polarization of mitochondrial distribution and directional cancer cell migration, which impact on cancer cell motility and extravasation.

## Discussion

Cell motility and migration is crucial for organism development, including organogenesis, normal growth, and repair such as wound healing. However, in inappropriate contexts, such as cancers, cell motility and migration can have devastating consequences. Metastasis constitutes the primary cause of death for >90% of patients with cancer (Steeg, 2006). Understanding the molecular players involved in this process should help identify targets for molecular therapies that can halt or even prevent cancer metastasis. The gene encoding SLC2A5, a fructose-specific transporter, is highly expressed in cancers whereas it is tightly regulated in healthy tissues (Douard & Ferraris, 2008). Specifically, increased abundance of both SLC2A5 mRNA and protein have been associated with cancer progression and increased frequency of metastasis of many cancers (Zamora-Leon et al., 1996; Chen et al., 2016; Bu et al., 2018; Hamann et al., 2018; Weng et al., 2018; Jin et al., 2019; Chen et al., 2020; Liang et al., 2021; Lin et al., 2021). In this study, we assessed the importance of the SLC2A5 gene on cancer cell proliferation, migration, extravasation, and colony formation. We found that CRISPR/Cas9-mediated inactivation of the SLC2A5 gene reduced cancer cell proliferation and inhibited motility in a variety of cancer cell lines. Specifically, the attenuation of the SLC2A5 gene expression inhibited cancer cell invasion and metastasis *in vivo* as we demonstrated in chick embryo, mouse, and zebrafish models. Furthermore, we discovered that suppression of the SLC2A5 gene in cancer cells resulted in notable changes in mitochondrial architecture and distribution, which substantially altered cell migration. Finally, our trans complementation experiments demonstrated that full activity of SLC2A5 is necessary for the enhanced proliferation and motility exhibited by cancer cells since the re-introduction of a mutant SLC2A5 defective for fructose binding/transport was unable to restore the phenotype of SLC2A5-attenuated cancer cells to that observed for cancer cells with wild-type SLC2A5.

Fructose can enter several important metabolic pathways critical for cancer growth including the hexosamine biosynthetic pathway (Chiaradonna et al., 2018), the pentose phosphate pathway (Stincone et al., 2015), and *de novo* lipogenesis (Ameer et al., 2014). Fructose can be metabolized to fatty acids and triglycerides, providing components essential for the synthesis of membrane lipids to sustain cancer growth and proliferation (Ter Horst and Serlie, 2017). Recent studies have also shown that fructose supplementation stimulates lung cancer cell proliferation *in vivo* (Chen et al., 2020; Liang et al., 2021). Accordingly, we found that supplementation of the cell culture medium with fructose robustly stimulated MIA-PaCa-2 cell proliferation while inactivation of the gene encoding SLC2A5 abolished this response. Curiously however, we observed that HT1080tdT cells, a fluorescently-tagged derivative of the highly tumorigenic and metastatic HT1080 fibrosarcoma (Gupta et al., 2001; Zuber et al., 2008; Castoria et al., 2013), did not respond to fructose supplementation as expected (Figure 3C). Although there was a tendency towards higher growth rate in response to fructose supplementation, the increment was not statistically significant. It is possible that HT1080 cells harbor additional pathways that enable them to grow more aggressively than other cancer cell lines. Regardless, inactivation of SLC2A5 did significantly decrease the HT1080 growth rate, suggesting that these cells can and do utilize fructose as a fuel source. Given that SLC2A5 is responsible for the import of fructose, our finding suggests that at least some of the fructose endogenously produced by HT1080 cells leave the cell and must re-enter via SLC2A5 before it can be used. The attenuation of cell proliferation as a consequence of SLC2A5 gene editing further supports the idea that HT1080 cells depend on fructose as a fuel.

One consequence of SLC2A5 inactivation common to both MIA-PaCa-2 and HT1080 cells relates to the remarkable alteration of mitochondrial distribution and morphology. Mitochondria are dynamic organelles that supply the energy required to drive the key cellular processes involved in metastasis, including proliferation and migration (Trotta & Chipuk, 2017). Critical to cancer cell proliferation and migration are changes in mitochondrial architecture, fusion, fission, and networking (Trotta & Chipuk, 2017). Mitochondria localize to the cell migratory front edge where they participate in podosome formation, and cell migration, invasion, and subsequently establish the metastatic site (Han et al., 2013; Landry et al., 2014; Leong et al., 2014; Stoletov & Lewis, 2015; Caswell & Zech, 2018; Denisenko et al., 2019; Furnish & Caino, 2020). During cancer cell migration, cells form directional invadopodia that ultimately penetrate the vascular wall (Stoletov et al., 2010; Stoletov et al., 2018). It is thought that mitochondria localized to these cellular protrusions provide the energy required for cellular movement and traversal of the vascular wall (Zhao et al., 2013; Caino et al., 2015; Caswell & Zech, 2018). The inhibition of the SLC2A5 gene resulted in changes in mitochondrial distribution and morphology that affected cellular migration. In SLC2A5-deficient cancer cells, mitochondria became elongated, increased in number, and dispersed throughout the cell, which prevented efficient cellular extravasation. This indicates that SLC2A5 function is required for mitochondrial polarization towards the cell protrusions and directional migration of cancer cells.

In conclusion, we demonstrated that limiting the function of the SLC2A5 (GLUT5) fructose transporter inhibited cell proliferation, motility and cancer cell metastasis. We also unexpectedly discovered that the localization and structure of mitochondria in cancer cells with attenuated SLC2A5 function contribute a role in the metastatic potential of cancer cells. Based on our findings, inhibition of SLC2A5 is a useful strategy for reducing the risk of metastasis, a deadly aspect of human cancers.

## Data Availability

The original contributions presented in the study are included in the article/Supplementary Materials, further inquiries can be directed to the corresponding authors.

## References

[B1] Ameer F. Scandiuzzi L. Hasnain S. Kalbacher H. Zaidi N. (2014). De novo lipogenesis in health and disease. Metabolism. 63, 895–902. 10.1016/j.metabol.2014.04.003 24814684

[B2] Borowicz S. Van Scoyk M. Avasarala S. Karuppusamy Rathinam M. K. Tauler J. Bikkavilli R. K. (2014). The soft agar colony formation assay. J. Vis. Exp. 92, e51998. 10.3791/51998 PMC4353381 25408172

[B3] Bu P. Chen K. Y. Xiang K. Johnson C. Crown S. B. Rakhilin N. (2018). Aldolase B-mediated fructose metabolism drives metabolic reprogramming of colon cancer liver metastasis. Cell Metab. 27, 1249–1262. e1244. 10.1016/j.cmet.2018.04.003 29706565 PMC5990465

[B4] Budczies J. von Winterfeld M. Klauschen F. Bockmayr M. Lennerz J. K. Denkert C. (2015). The landscape of metastatic progression patterns across major human cancers. Oncotarget 6, 570–583. 10.18632/oncotarget.2677 25402435 PMC4381616

[B5] Caino M. C. Ghosh J. C. Chae Y. C. Vaira V. Rivadeneira D. B. Faversani A. (2015). PI3K therapy reprograms mitochondrial trafficking to fuel tumor cell invasion. Proc. Natl. Acad. Sci. U. S. A. 112, 8638–8643. 10.1073/pnas.1500722112 26124089 PMC4507184

[B6] Castoria G. Giovannelli P. Di Donato M. Hayashi R. Arra C. Appella E. (2013). Targeting androgen receptor/Src complex impairs the aggressive phenotype of human fibrosarcoma cells. PLoS One 8, e76899. 10.1371/journal.pone.0076899 24130806 PMC3793924

[B7] Caswell P. T. Zech T. (2018). Actin-based cell protrusion in a 3D matrix. Trends Cell Biol. 28, 823–834. 10.1016/j.tcb.2018.06.003 29970282 PMC6158345

[B8] Chaffer C. L. Weinberg R. A. (2011). A perspective on cancer cell metastasis. Science 331, 1559–1564. 10.1126/science.1203543 21436443

[B9] Chen W. L. Jin X. Wang M. Liu D. Luo Q. Tian H. (2020). GLUT5-mediated fructose utilization drives lung cancer growth by stimulating fatty acid synthesis and AMPK/mTORC1 signaling. JCI Insight 5, e131596. 10.1172/jci.insight.131596 PMC7098789 32051337

[B10] Chen W. L. Wang Y. Y. Zhao A. Xia L. Xie G. Su M. (2016). Enhanced fructose utilization mediated by SLC2A5 is a unique metabolic feature of acute myeloid leukemia with therapeutic potential. Cancer Cell 30, 779–791. 10.1016/j.ccell.2016.09.006 27746145 PMC5496656

[B11] Chiaradonna F. Ricciardiello F. Palorini R. (2018). The nutrient-sensing hexosamine biosynthetic pathway as the hub of cancer metabolic rewiring. Cells 7, 53. 10.3390/cells7060053 PMC6025041 29865240

[B12] Denisenko T. V. Gorbunova A. S. Zhivotovsky B. (2019). Mitochondrial involvement in migration, invasion and metastasis. Front. Cell Dev. Biol. 7, 355. 10.3389/fcell.2019.00355 31921862 PMC6932960

[B13] Desai S. P. Bhatia S. N. Toner M. Irimia D. (2013). Mitochondrial localization and the persistent migration of epithelial cancer cells. Biophys. J. 104, 2077–2088. 10.1016/j.bpj.2013.03.025 23663851 PMC3647149

[B14] Douard V. Ferraris R. P. (2008). Regulation of the fructose transporter GLUT5 in health and disease. Am. J. Physiol. Endocrinol. Metab. 295, E227–E237. 10.1152/ajpendo.90245.2008 18398011 PMC2652499

[B15] Furnish M. Caino M. C. (2020). Altered mitochondrial trafficking as a novel mechanism of cancer metastasis. Cancer Rep. 3, e1157. 10.1002/cnr2.1157 PMC7370516 32671955

[B16] Groenendyk J. Peng Z. Dudek E. Fan X. Mizianty M. J. Dufey E. (2014). Interplay between the oxidoreductase PDIA6 and microRNA-322 controls the response to disrupted endoplasmic reticulum calcium homeostasis. Sci. Signal. 7, ra54. 10.1126/scisignal.2004983 24917591 PMC4984425

[B17] Gupta S. Stuffrein S. Plattner R. Tencati M. Gray C. Whang Y. E. (2001). Role of phosphoinositide 3-kinase in the aggressive tumor growth of HT1080 human fibrosarcoma cells. Mol. Cell. Biol. 21, 5846–5856. 10.1128/mcb.21.17.5846-5856.2001 11486024 PMC87304

[B18] Hamann I. Krys D. Glubrecht D. Bouvet V. Marshall A. Vos L. (2018). Expression and function of hexose transporters GLUT1, GLUT2, and GLUT5 in breast cancer-effects of hypoxia. FASEB J. 32, 5104–5118. 10.1096/fj.201800360R 29913554

[B19] Han T. Kang D. Ji D. Wang X. Zhan W. Fu M. (2013). How does cancer cell metabolism affect tumor migration and invasion? Cell adh. Migr. 7, 395–403. 10.4161/cam.26345 24131935 PMC3903682

[B20] Jin C. Gong X. Shang Y. (2019). GLUT5 increases fructose utilization in ovarian cancer. Onco. Targets. Ther. 12, 5425–5436. 10.2147/OTT.S205522 31371983 PMC6635899

[B21] Landry M. C. Champagne C. Boulanger M. C. Jette A. Fuchs M. Dziengelewski C. (2014). A functional interplay between the small GTPase Rab11a and mitochondria-shaping proteins regulates mitochondrial positioning and polarization of the actin cytoskeleton downstream of Src family kinases. J. Biol. Chem. 289, 2230–2249. 10.1074/jbc.M113.516351 24302731 PMC3900968

[B22] Legros F. Lombes A. Frachon P. Rojo M. (2002). Mitochondrial fusion in human cells is efficient, requires the inner membrane potential, and is mediated by mitofusins. Mol. Biol. Cell 13, 4343–4354. 10.1091/mbc.e02-06-0330 12475957 PMC138638

[B23] Leong H. S. Robertson A. E. Stoletov K. Leith S. J. Chin C. A. Chien A. E. (2014). Invadopodia are required for cancer cell extravasation and are a therapeutic target for metastasis. Cell Rep. 8, 1558–1570. 10.1016/j.celrep.2014.07.050 25176655

[B24] Liang R. J. Taylor S. Nahiyaan N. Song J. Murphy C. J. Dantas E. (2021). GLUT5 (SLC2A5) enables fructose-mediated proliferation independent of ketohexokinase. Cancer Metab. 9, 12. 10.1186/s40170-021-00246-9 33762003 PMC7992954

[B25] Lin M. Fang Y. Li Z. Li Y. Feng X. Zhan Y. (2021). S100P contributes to promoter demethylation and transcriptional activation of SLC2A5 to promote metastasis in colorectal cancer. Br. J. Cancer 125, 734–747. 10.1038/s41416-021-01306-z 34188196 PMC8405647

[B26] Nomura N. Verdon G. Kang H. J. Shimamura T. Nomura Y. Sonoda Y. (2015). Structure and mechanism of the mammalian fructose transporter GLUT5. Nature 526, 397–401. 10.1038/nature14909 26416735 PMC4618315

[B27] Prins D. Groenendyk J. Touret N. Michalak M. (2011). Modulation of STIM1 and capacitative Ca^2+^ entry by the endoplasmic reticulum luminal oxidoreductase ERp57. EMBO Rep. 12, 1182–1188. 10.1038/embor.2011.173 21941299 PMC3207099

[B28] Rasheed S. Nelson-Rees W. A. Toth E. M. Arnstein P. Gardner M. B. (1974). Characterization of a newly derived human sarcoma cell line (HT-1080). Cancer 33, 1027–1033. 10.1002/1097-0142(197404)33:4<1027::aid-cncr2820330419>3.0.co;2-z 4132053

[B29] Schuler M. H. Lewandowska A. Caprio G. D. Skillern W. Upadhyayula S. Kirchhausen T. (2017). Miro1-mediated mitochondrial positioning shapes intracellular energy gradients required for cell migration. Mol. Biol. Cell 28, 2159–2169. 10.1091/mbc.E16-10-0741 28615318 PMC5531732

[B30] Steeg P. S. (2016). Targeting metastasis. Nat. Rev. Cancer 16, 201–218. 10.1038/nrc.2016.25 27009393 PMC7055530

[B31] Steeg P. S. (2006). Tumor metastasis: Mechanistic insights and clinical challenges. Nat. Med. 12, 895–904. 10.1038/nm1469 16892035

[B32] Stincone A. Prigione A. Cramer T. Wamelink M. M. Campbell K. Cheung E.. (2015). The return of metabolism: Biochemistry and physiology of the pentose phosphate pathway. Biol. Rev. Camb. Philos. Soc. 90, 927–963. 10.1111/brv.12140 25243985 PMC4470864

[B33] Stoletov K. Kato H. Zardouzian E. Kelber J. Yang J. Shattil S. (2010). Visualizing extravasation dynamics of metastatic tumor cells. J. Cell Sci. 123, 2332–2341. 10.1242/jcs.069443 20530574 PMC2886748

[B34] Stoletov K. Lewis J. D. (2015). Invadopodia: A new therapeutic target to block cancer metastasis. Expert Rev. Anticancer Ther. 15, 733–735. 10.1586/14737140.2015.1058711 26098830

[B35] Stoletov K. Montel V. Lester R. D. Gonias S. L. Klemke R. (2007). High-resolution imaging of the dynamic tumor cell vascular interface in transparent zebrafish. Proc. Natl. Acad. Sci. U. S. A. 104, 17406–17411. 10.1073/pnas.0703446104 17954920 PMC2077269

[B36] Stoletov K. Willetts L. Paproski R. J. Bond D. J. Raha S. Jovel J. (2018). Quantitative *in vivo* whole genome motility screen reveals novel therapeutic targets to block cancer metastasis. Nat. Commun. 9, 2343. 10.1038/s41467-018-04743-2 29904055 PMC6002534

[B37] Strilic B. Offermanns S. (2017). Intravascular survival and extravasation of tumor cells. Cancer Cell 32, 282–293. 10.1016/j.ccell.2017.07.001 28898694

[B38] Sun X. Cao H. Zhan L. Yin C. Wang G. Liang P. (2018). Mitochondrial fission promotes cell migration by Ca^2+^/CaMKII/ERK/FAK pathway in hepatocellular carcinoma. Liver Int. 38, 1263–1272. 10.1111/liv.13660 29210177

[B39] Ter Horst K. W. Serlie M. J. (2017). Fructose consumption, lipogenesis, and non-alcoholic fatty liver disease. Nutrients 9, 981. 10.3390/nu9090981 PMC5622741 28878197

[B40] Trotta A. P. Chipuk J. E. (2017). Mitochondrial dynamics as regulators of cancer biology. Cell. Mol. Life Sci. 74, 1999–2017. 10.1007/s00018-016-2451-3 28083595 PMC5419868

[B41] Uldry M. Thorens B. (2004). The SLC2 family of facilitated hexose and polyol transporters. Pflugers Arch. 447, 480–489. 10.1007/s00424-003-1085-0 12750891

[B42] Weng Y. Zhu J. Chen Z. Fu J. Zhang F. (2018). Fructose fuels lung adenocarcinoma through GLUT5. Cell Death Dis. 9, 557. 10.1038/s41419-018-0630-x 29748554 PMC5945656

[B43] Willetts L. Bond D. Stoletov K. Lewis J. D. (2016). Quantitative analysis of human cancer cell extravasation using intravital imaging. Methods Mol. Biol. 1458, 27–37. 10.1007/978-1-4939-3801-8_3 27581012

[B44] Yunis A. A. Arimura G. K. Russin D. J. (1977). Human pancreatic carcinoma (MIA PaCa-2) in continuous culture: Sensitivity to asparaginase. Int. J. Cancer 19, 128–135. 10.1002/ijc.2910190118 832918

[B45] Zamora-Leon S. P. Golde D. W. Concha II Rivas C. I. Delgado-Lopez F. Baselga J. (1996). Expression of the fructose transporter GLUT5 in human breast cancer. Proc. Natl. Acad. Sci. U. S. A. 93, 1847–1852. 10.1073/pnas.93.5.1847 8700847 PMC39870

[B46] Zhao J. Zhang J. Yu M. Xie Y. Huang Y. Wolff D. W. (2013). Mitochondrial dynamics regulates migration and invasion of breast cancer cells. Oncogene 32, 4814–4824. 10.1038/onc.2012.494 23128392 PMC3911914

[B47] Zuber C. Knackmuss S. Zemora G. Reusch U. Vlasova E. Diehl D. (2008). Invasion of tumorigenic HT1080 cells is impeded by blocking or downregulating the 37-kDa/67-kDa laminin receptor. J. Mol. Biol. 378, 530–539. 10.1016/j.jmb.2008.02.004 18387633

