# Supplementary material for: Loss of the fructose transporter SLC2A5 inhibits cancer cell migration
Source: Front Cell Dev Biol. 2022 Sep 30;10:896297. doi: 10.3389/fcell.2022.896297 (PMC9578049; doi:10.3389/fcell.2022.896297)
Supplement: Supplementary file 1 [file DataSheet7.PDF]

LEGENDS TO THE SUPPLEMENTAL VIDEOS

Supplemental Video S1. Time lapse video of metastatic behaviour of red fluorescent protein-labelled HT1080tdT and HT1080tdT-ε2A5 using chicken embryo chorioallantoic membrane (CAM) system.

Supplemental Video S2. Time lapse video of mitochondria (green) movement in red fluorescent protein-labelled HT1080tdT and HT1080tdT-ε2A5 cells. The cell nuclei are stained with DAPI (blue). Note the area of within HT1080tdT cells (e.g., arrowhead) where a high concentration of mitochondria are rapidly migrating toward the leading edge of movement.

Supplemental Video S3. Time lapse images of mitochondria movement (green) in red fluorescent protein-labelled HT1080tdT and HT1080tdT-ε2A5 cells extravasating into the chicken embryo chorioallantoic membrane (CAM) vasculature.
