# Supplementary material for: Loss of the fructose transporter SLC2A5 inhibits cancer cell migration
Source: Front Cell Dev Biol. 2022 Sep 30;10:896297. doi: 10.3389/fcell.2022.896297 (PMC9578049; doi:10.3389/fcell.2022.896297)
Supplement: Supplementary file 2 [file DataSheet2.PDF]

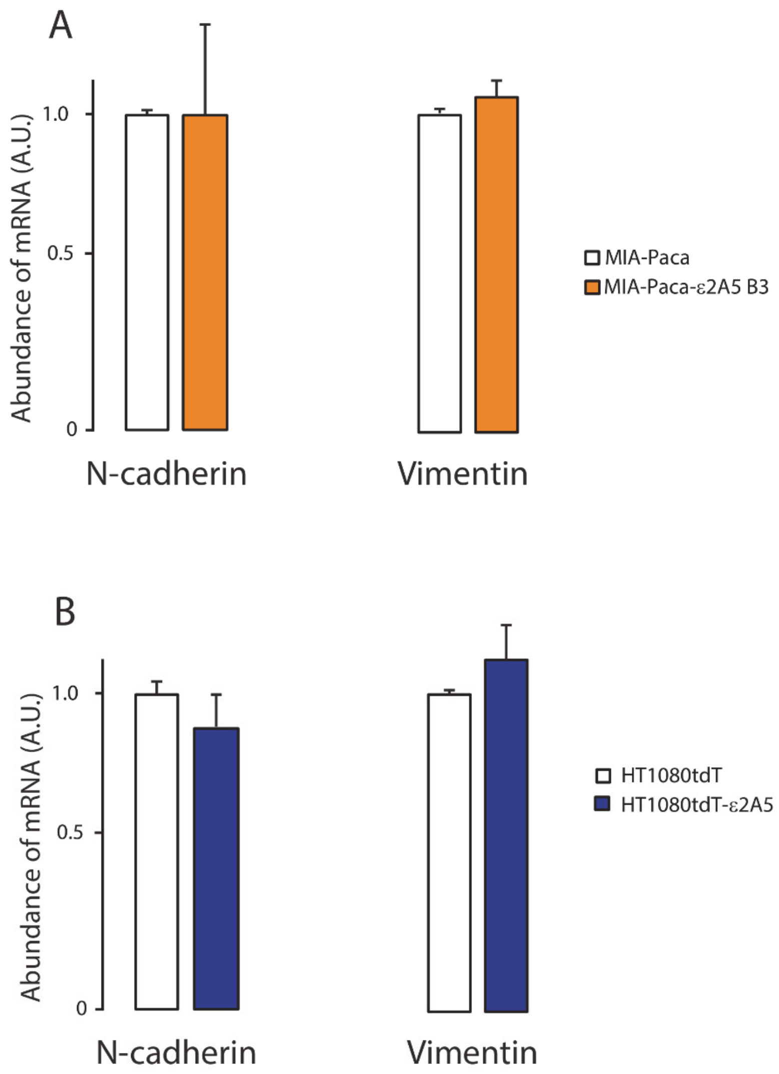

Supplemental Figure S2. Analysis of the abundance of N-cadherin and vimentin. A. Abundance of mRNA encoding N-cadherin and vimentin in MIA-PaCa-2 and MIA-PaCa-ε2A5 B3 cell lines. (n=3) **B.** qPCR analysis of N-cadherin and vimentin mRNA in HT1080tdT and HT1080tdT-ε2A5 cells. (n=3)
