# Supplementary material for: Loss of the fructose transporter SLC2A5 inhibits cancer cell migration
Source: Front Cell Dev Biol. 2022 Sep 30;10:896297. doi: 10.3389/fcell.2022.896297 (PMC9578049; doi:10.3389/fcell.2022.896297)
Supplement: Supplementary file 3 [file DataSheet4.PDF]

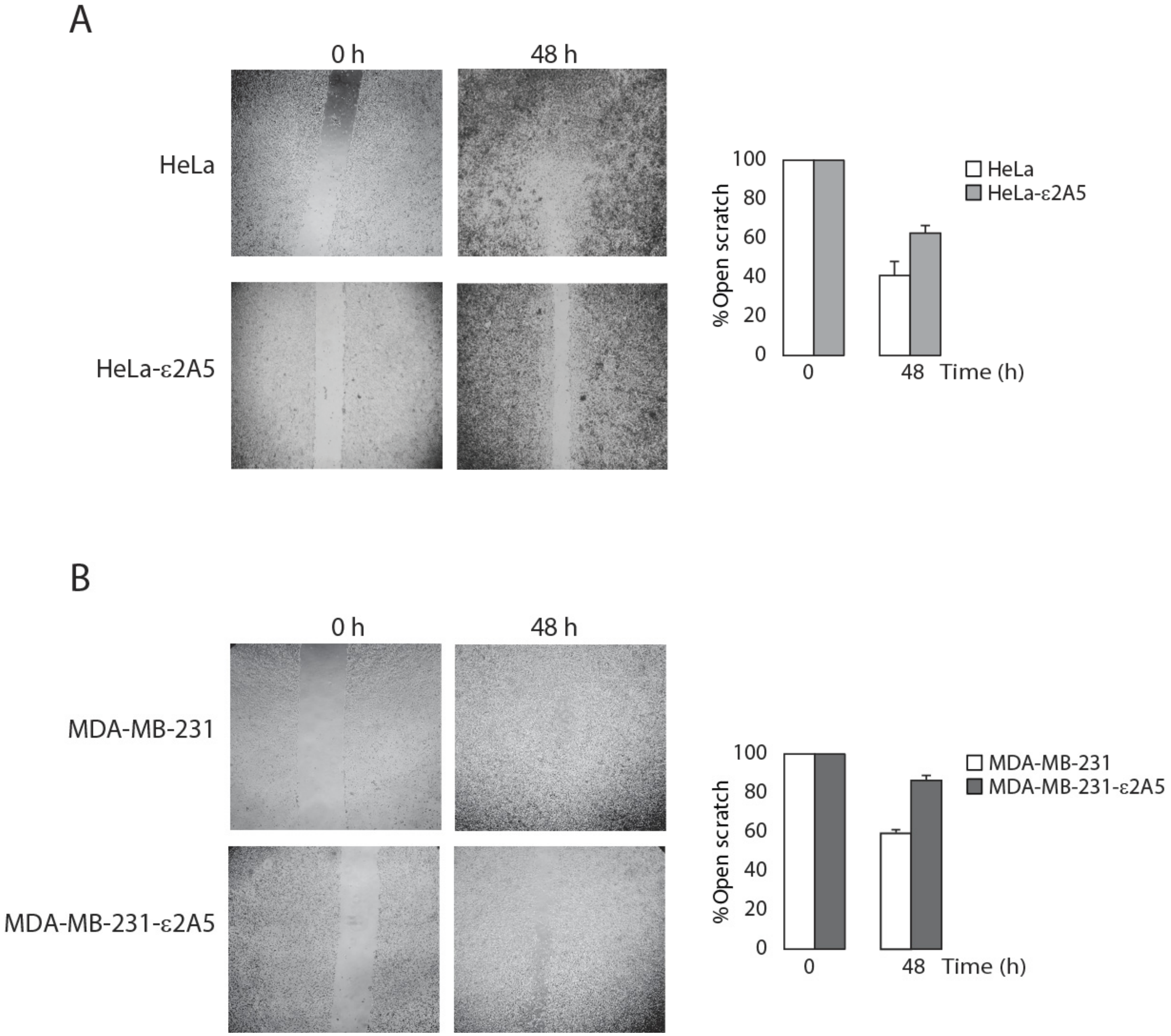

Supplemental Figure S4. Scratch test of the HeLa and MDA-MB-231 cells. Images of scratch test (left) and quantitative analysis of closure (right) as a function of time of **A.** HeLa and HeLa HeLa-ε2A5, and **B.** MDA-MB-231 and MDA-MB-231-ε2A5 cells. **p*=0.0003; ***p*=0.0001 (n=3).
