# Supplementary material for: Loss of the fructose transporter SLC2A5 inhibits cancer cell migration
Source: Front Cell Dev Biol. 2022 Sep 30;10:896297. doi: 10.3389/fcell.2022.896297 (PMC9578049; doi:10.3389/fcell.2022.896297)
Supplement: Supplementary file 4 [file DataSheet6.PDF]

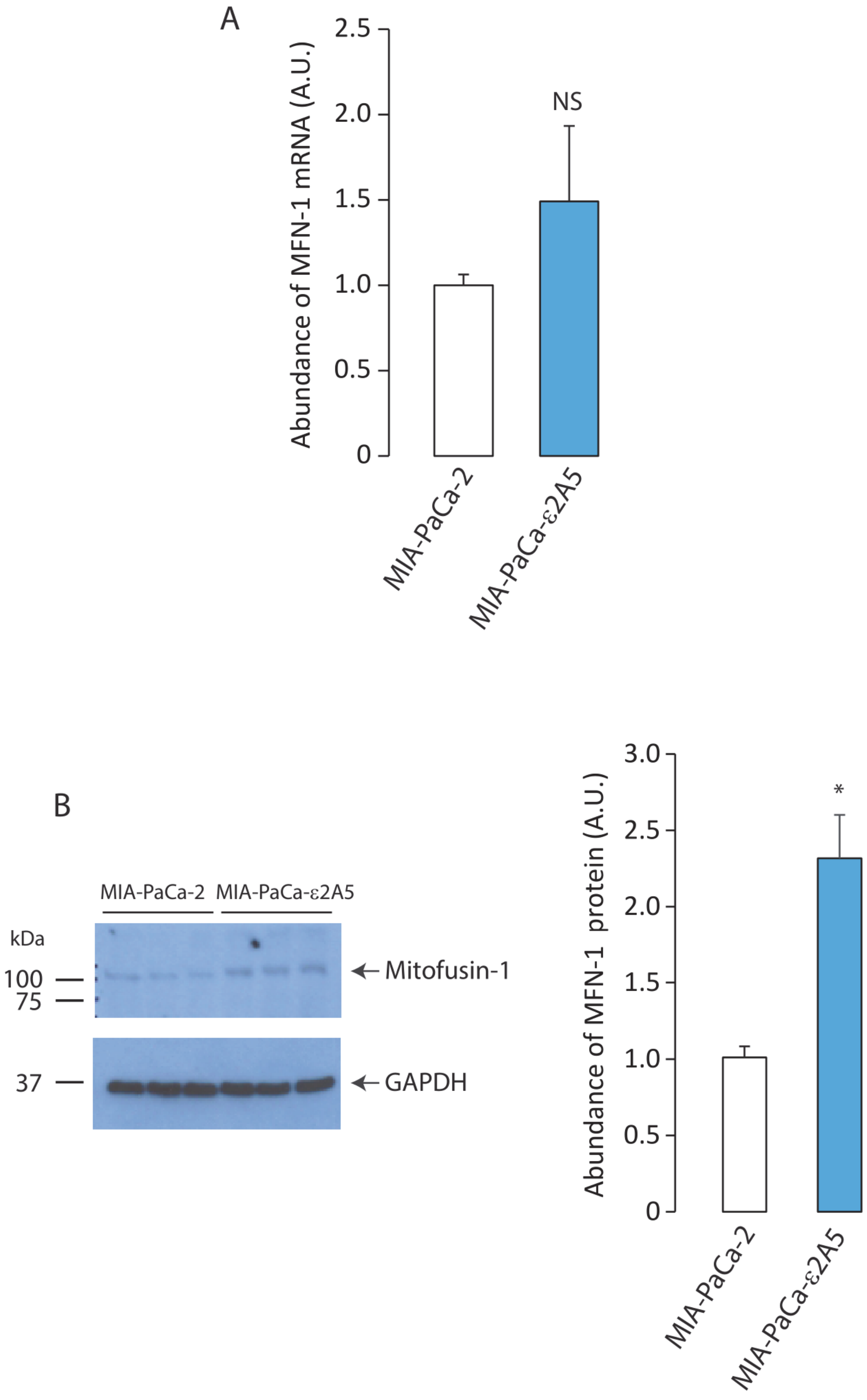

Supplemental Figure S6. Mitofusin-1 mRNA and protein in MIA-PaCa-2 and MIA-PaCa-ε2A5 cells. A. qPCR analysis of the abundance of mitofusin-1 (MFN-1) mRNA. **B.** Immunoblot was probed with anti-mitofusin-1 antibodies followed by quantitative analysis of protein bands (right graph). Anti-GAPDH antibodies were used to assess protein loading. ***p* <0.011; n=3; NS, not significant. All data in the Figure is representative of more than 3 biological replicates.
