# Supplementary material for: Loss of the fructose transporter SLC2A5 inhibits cancer cell migration
Source: Front Cell Dev Biol. 2022 Sep 30;10:896297. doi: 10.3389/fcell.2022.896297 (PMC9578049; doi:10.3389/fcell.2022.896297)
Supplement: Supplementary file 6 [file DataSheet3.PDF]

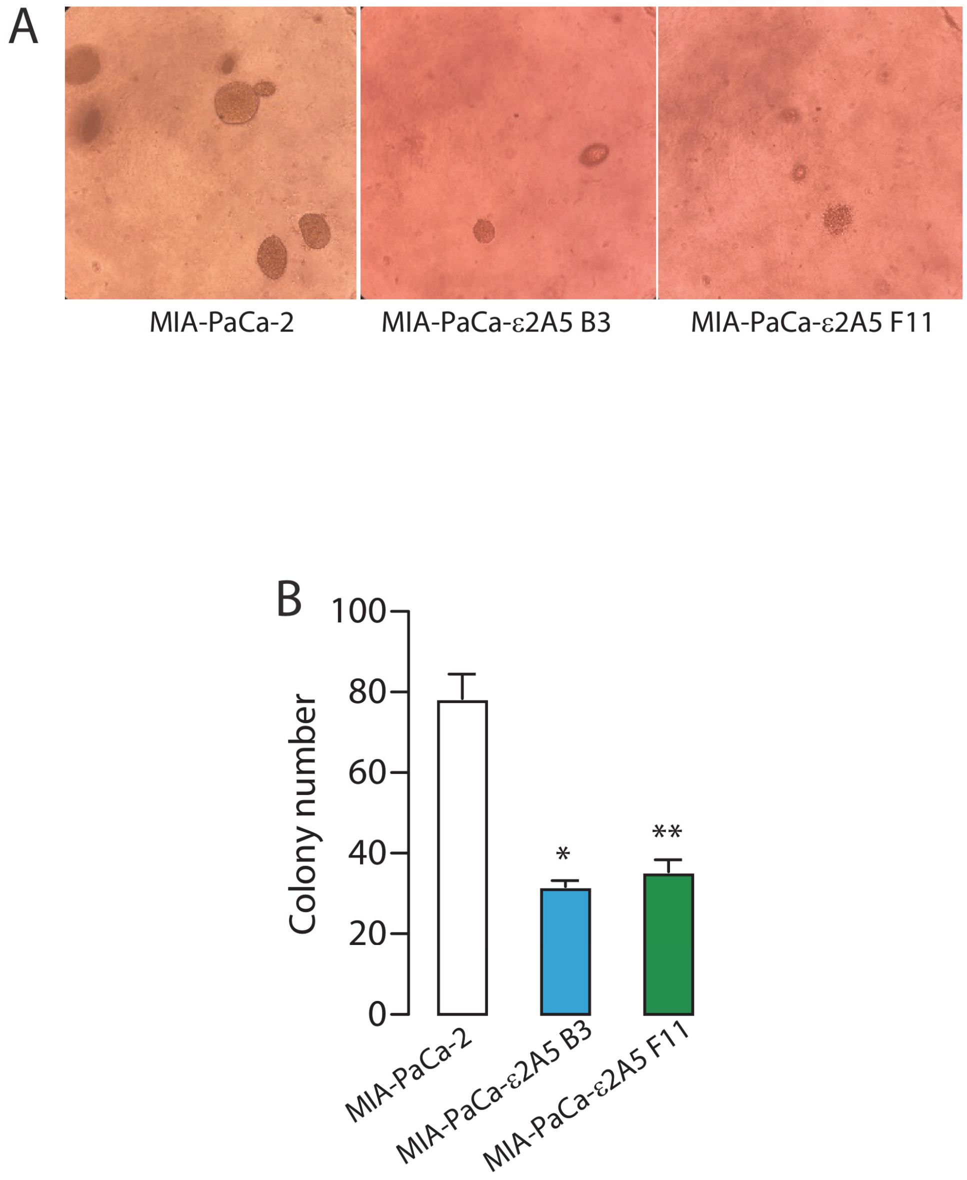

Supplemental Figure S3. Colony formation in the soft agar transformation assay. A. Images of colonies formed in 0.6% agarose by MIA-PaCa-2 and MIA-PaCa-ε2A5 cell lines (clones B3 and F11). **B.** Analysis of colony number for MIA-PaCa-2 and MIA-PaCa-ε2A5 cell lines (clones B3 and F11). **p*=0.0002; ***p*=0.0004; n=3. The images are representative of more than 3 biological replicates.
