# Supplementary material for: Loss of the fructose transporter SLC2A5 inhibits cancer cell migration
Source: Front Cell Dev Biol. 2022 Sep 30;10:896297. doi: 10.3389/fcell.2022.896297 (PMC9578049; doi:10.3389/fcell.2022.896297)
Supplement: Supplementary file 7 [file DataSheet1.PDF]

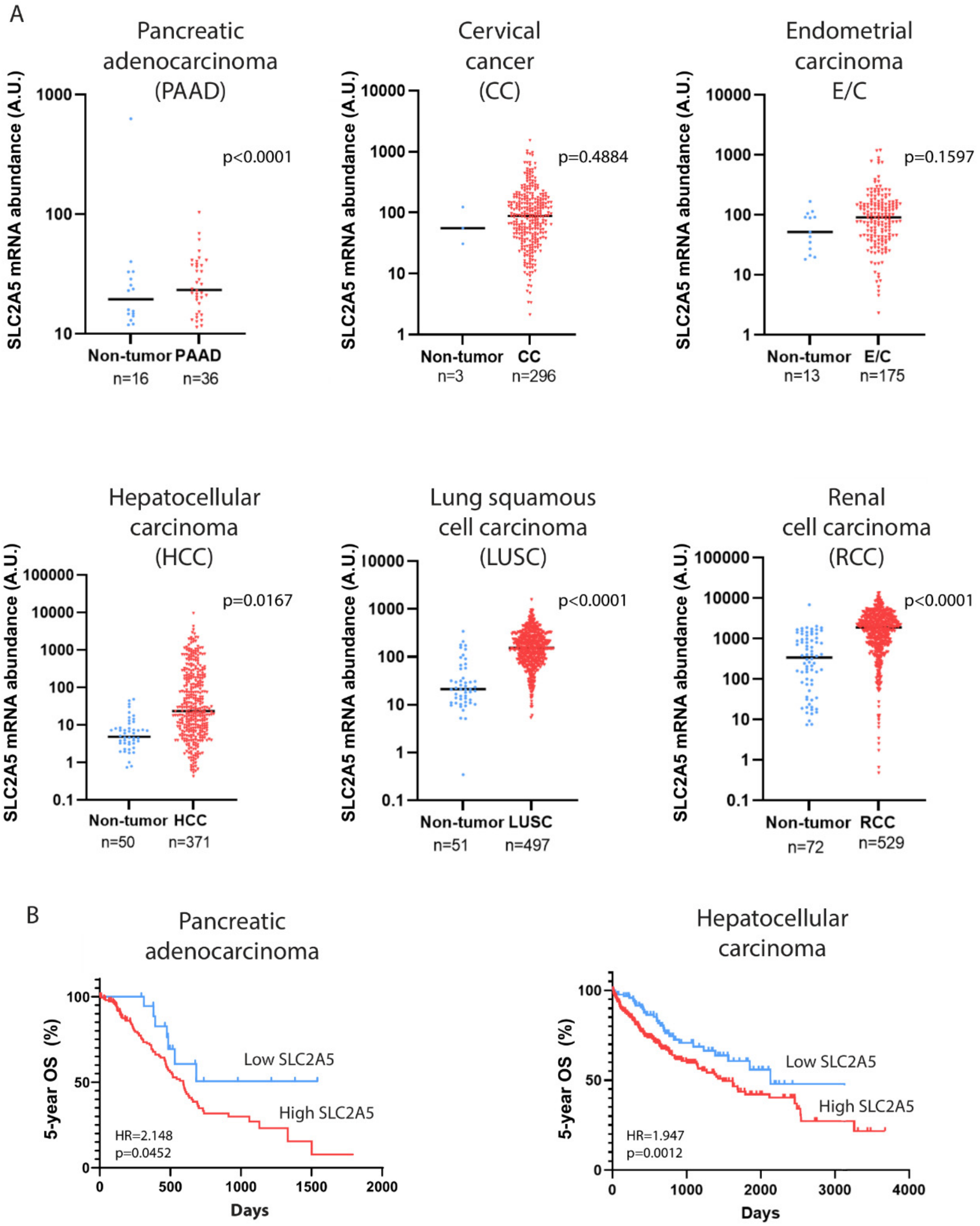

Supplemental Figure S1. Public TCGA dataset analysis of SLC2A5 association with increased progression and metastasis of selected human cancers. The Cancer Genome Atlas (TCGA) Liver Heptaocelluar (LIHC) and Pancreatic Adenocarcinoma (PAAD) human cancer RNA-Seq data were obtained from the Genomic Data Commons Data Portal (https://portal.gdc.cancer.gov/). Primary tumor samples were used in this analysis. Overall Survival (OS) data from the TCGA LIHC and PAAD cohort was obtained from the integrated TCGA Pan-cancer clinical data resource. OS was defined as death from any cause from the date of diagnosis. The survival package of R 3.5.0 was used to perform survival analysis. 5-year OS was evaluated by Kaplan–Meier survival analysis and the log-rank test. LIHC or PAAD cases were divided into two groups with high and low SLC2A5 expression. All expression values from the 10th to 90th percentiles were used to group the cases, examine significant level differences in the OS of the groups, and the value yielding the lowest log-rank *p*-values was selected. Hazard ratio (HR) > 1 and $p < 0.05$ in the five-year was considered significant. **A**. TCGA LIHC and PAAD RNA-Seq data for human cervical cancer, renal carcinoma, lung carcinoma, hepatocellular carcinoma, and pancreatic cancer were obtained from the Genomic Data Commons Data Portal (https://portal.gdc.cancer.gov and https://www.cancer.gov). The value yielding the lowest log-rank *p*-values was selected. **B.** Kaplan–Meier survival analysis and log-rank test for pancreatic adenocarcinoma and hepatocellular carcinoma. Hazard ratio (HR) > 1 and $p < 0.05$ in the five-year was considered significant. OS, overall survival.
