# Supplementary material for: Loss of the fructose transporter SLC2A5 inhibits cancer cell migration
Source: Front Cell Dev Biol. 2022 Sep 30;10:896297. doi: 10.3389/fcell.2022.896297 (PMC9578049; doi:10.3389/fcell.2022.896297)
Supplement: Supplementary file 8 [file DataSheet5.PDF]

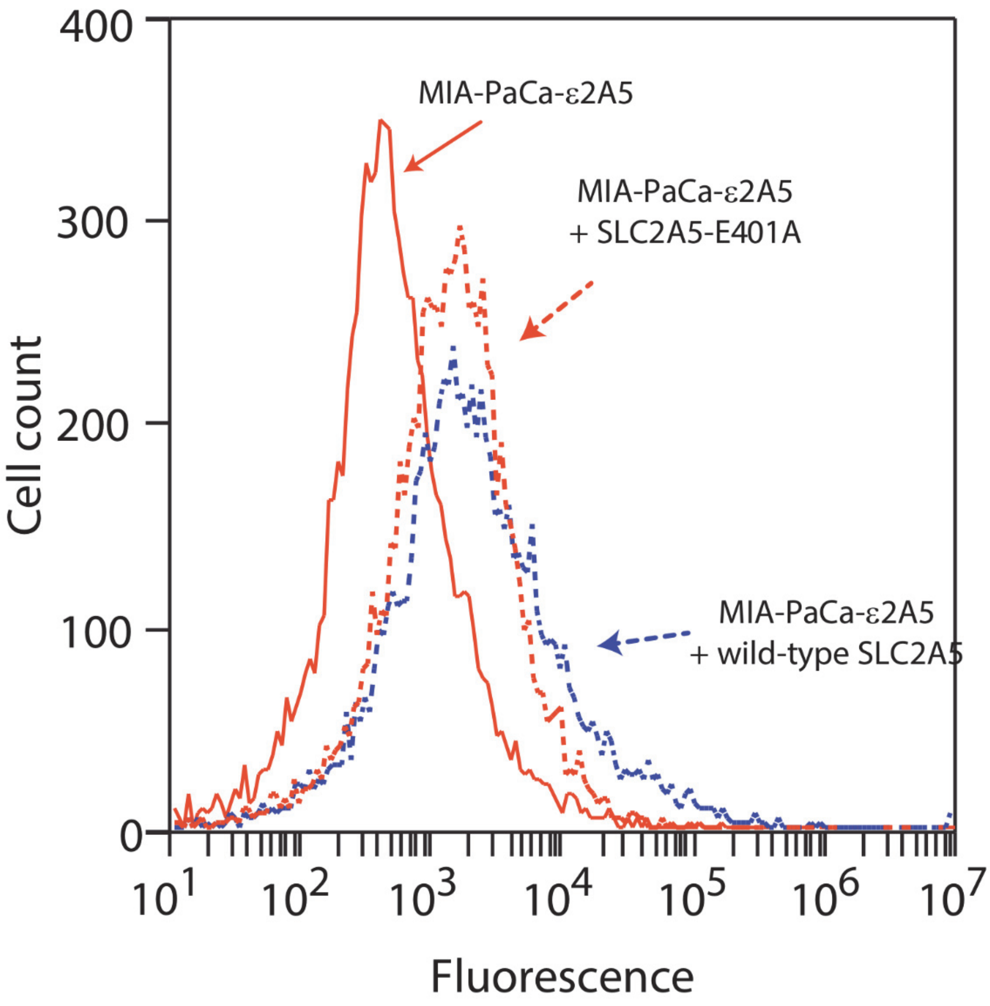

Supplemental Figure S5. Flow Cytometry analysis MIA-PaCa-ε2A5 cells. Representative histograms from flow cytometry analysis of MIA-PaCa-ε2A5 (clone F11) or MIA-PaCa-ε2A5 (clone F11) cells transfected with expression vector encoding Flag-tagged wild-type human SLC2A5 or SLC2A5 E401A mutant with anti-Flag antibodies. Solid orange line represents MIA-PaCa-ε2A5, dashed orange line MIA-PaCa-ε2A5 expressing the non-functional SLC2A5-E401A mutant; blue dashed line MIA-PaCa-ε2A5 expressing wild-type SLC2A5.
